# Nox3 expression and function in retinal ganglion cells and Amacrine cells

**DOI:** 10.1007/s00018-025-05876-6

**Published:** 2025-12-03

**Authors:** Takehiko Ueyama, Kyoko Yamaguchi, Yakumo Aoyama, Kota Aoshima, Michiho Onizuka, Taichi Tamagawa, Shota Kitayama, Junichi Ueyama, Kiyoki Okamoto, Hiroaki Mohri, Masamitsu Shimazawa

**Affiliations:** 1 Laboratory of Molecular Pharmacology, Biosignal Research Center, Kobe University, Kobe, 657-8501 Japan; 2 Molecular Pharmacology, Department of Biofunctional Evaluation, Gifu Pharmaceutical University, Gifu, 501-1196 Japan; 3 1-1 Rokkodai-cho, Nada-ku, Kobe, 657-8501 Japan

**Keywords:** Aging, Cisplatin, Electroretinogram (ERG), NADPH oxidase (Nox), Optic nerve, Reactive oxygen species (ROS)

## Abstract

NADPH oxidase 3 (Nox3), expressed in the endolymphatic duct and sac within the vestibule of the inner ear, is essential for otoconia formation. Mice with functionally deficient Nox3 exhibit a “head-tilt” phenotype. Recently, we reported that Nox3 expression in the cochlea is induced by aging, cisplatin treatment, and intense noise exposure, contributing to the primary source of reactive oxygen species (ROS) and causing acquired sensorineural hearing loss. However, its expression and function outside the inner ear remain poorly characterized. To explore novel Nox3 functions, we used *Nox3-Cre;tdTomato* mice, in which Cre recombinase (*Cre*) is knocked into the *ATG* site of *Nox3*, enabling visualization of Nox3 expression via tdTomato fluorescence. We identified Nox3 expression in retinal ganglion cells (RGCs) and GABAergic amacrine cells (ACs). The tdTomato-positive cells increased by 2 months of age and then plateaued in *Nox3-Cre*^*+/−*^;*tdTomato*^*+/+*^ (heterozygous knock-in [KI], hereafter HT *Nox3*-knockout [KO]) mice, while in *Nox3-Cre*^*+/+*^;*tdTomato*^*+/+*^ (homozygous KI, hereafter *Nox3*-KO) mice, the increase occurred by 12 months, suggesting that Nox3-derived ROS are toxic to RGCs and ACs. Additionally, *Nox3*-KO mice showed reduced a-, b-, and scotopic threshold response (STR)-waves in electroretinogram (ERG) compared to WT mice. Treatment with cisplatin, a well-known toxic agent for the retina and optic nerve, reduced tdTomato-positive cells in the retinae of 2-month-old HT *Nox3-*KO mice but not in *Nox3-*KO mice, compared to age-matched controls. *Nox3*-KO retinae developed normally. Thus, Nox3 is expressed in RGCs and ACs to regulate retinal function; however, excessive Nox3-derived ROS cause toxicity under pathological conditions, including aging.

**Supplementary Information:**

The online version contains supplementary material available at 10.1007/s00018-025-05876-6.

## Introduction

NADPH oxidases are major sources of reactive oxygen species (ROS) in various cells and tissues [1]. They are the only enzyme family that generates ROS as their primary function [2]. Humans possess seven Nox isoforms (Nox1–5, Duox1–2) [1, 3]. Although appropriate ROS levels are indispensable for cell survival and differentiation [1], higher levels induce pathologies such as fibrosis, inflammation, cancer, and cardiovascular and neurodegenerative disorders [4, 5].

Nox3 was initially identified as essential for otoconia formation, with functionally deficient Nox3 mice exhibiting a “head-tilt” phenotype [6, 7]. Later studies confirmed that Nox3 is expressed in the endolymphatic duct and sac for otoconia biosynthesis [8]. Nox3, a membrane-bound heterodimer with p22^*phox*^, exhibits low levels of constitutive ROS production without stimulation or activator/organizer [5, 9], and shows enhanced activation with support of cytoplasmic factors (called organizers or activators), such as Noxo1/p47^*phox*^, Noxa1/p67^*phox*^, and Rac small GTPases [5, 9]. However, the mechanism of Nox3 activation differs between humans and mice: full activation of human Nox3 requires Noxo1 alone or p47^*phox*^ and p67^*phox*^ [9], whereas mouse Nox3 requires Noxo1/p47^*phox*^ and Noxa1/p67^*phox*^ for full activation [10]. Nox3 primarily generates superoxide, releasing it into the extracellular space from the plasma membrane [1, 3, 5, 9]. More recently, using *Nox3-Cre;tdTomato* mice (Fig. S1), where tdTomato is expressed under the control of the Nox3 promoter and its fluorescence marks cells with capabilities of Nox3 expression, we identified Nox3 expression in the cochlea, in addition to the endolymphatic duct and sac [7]. Furthermore, we reported that Nox3 expression in the cochlea is upregulated by aging, cisplatin treatment, and intense noise exposure, contributing to increased ROS production and causing acquired sensorineural hearing loss (SNHL) [7]. Since Nox inhibitors are promising candidates for treating ROS-related disorders, they have been intensively developed over the last two decades [4, 5]. Several compounds inhibiting Nox1, Nox2, and Nox4 are undergoing clinical trials; however, the development of Nox3 inhibitors lags the most [11] due to limited information on Nox3-related functions and disorders, as well as differences in activation mechanisms between humans and mice.

Although Nox3 has been described as the inner ear-specific Nox since its discovery [5], it has also been detected in several immortalized cell lines beyond the inner ear, including adipocytes [12], hepatocytes [13], and cancer cells [5]. However, reports showing protein expression and/or functional involvement of Nox3 at the individual level are limited to spermatogonia in the testis [14], endothelial cells in the lung [15], monocytes [16], and cerebellar neural progenitor cells [17]. Thus, Nox3 expression and function outside the inner ear and the reported cell types remain poorly characterized due to the lack of a reliable anti-Nox3 antibody.

To explore novel Nox3-expressing cells and their functions, *Nox3-Cre;tdTomato*^*+/+*^ mice were used (Fig. S1). Nox3 is expressed in retinal ganglion cells (RGCs) and amacrine cells (ACs). *Nox3-Cre*^*+/+*^;*tdTomato*^*+/+*^ (homozygous knock-in [KI], hereafter *Nox3*-knockout [KO]) mice showed reduced a-, b-, and scotopic threshold response (STR)-waves in the electroretinogram (ERG) compared to control mice. Additionally, cisplatin treatment decreased tdTomato-positive cells in *Nox3-Cre*^*+/−*^;*tdTomato*^*+/+*^ (heterozygous KI, hereafter HT *Nox3*-KO) mice but not in *Nox3*-KO mice. Thus, Nox3 expressed in RGCs and ACs regulates retinal function; however, Nox3 exerts harmful effects under pathological conditions in the retina, including aging and cisplatin treatment.

## Materials and methods

### Animals

*Nox3-Cre;tdTomato* mice on a C57BL/6J background were generated by crossing *Nox3-Cre* KI mice [7], in which the Cre recombinase encoding gene (*Cre*) is inserted into the *ATG* site of exon 1 of *Nox3*, with *CAG-STOP*^*flox*^*-tdTomato* (Ai9) reporter mice, where tdTomato is inserted into the ROSA26 locus (obtained from Jackson Laboratory, Bar Harbor, ME, USA). This crossing produced *Nox3-Cre*^*−/−*^;*tdTomato*^*+/+*^, *Nox3-Cre*^*+/−*^;*tdTomato*^*+/+*^, *Nox3-Cre*^*+/+*^;*tdTomato*^*+/+*^ mice (Fig. S1). In the latter two lines, *Nox3-promote*r-driven Cre expression induced tdTomato fluorescence in potentially Nox3-expressing cells. The *Nox3-Cre*^*−/−*^;*tdTomato*^*+/+*^, *Nox3-Cre*^*+/−*^;*tdTomato*^*+/+*^, and *Nox3-Cre*^*+/+*^;*tdTomato*^*+/+*^ lines were used as WT (or control), HT *Nox3*-KO, and *Nox3*-KO mice, respectively.

The offspring of these mice were genotyped by PCR using the following primer pair sets: 5′-CTTGGCACTAAGTCCTTGATTAG-3′ and 5′-CAGTGAAACAGCATTGCTGTC-3′ for *Nox3-Cre*; 5′-CTGTTCCTGTACGGCATGG-3′ and 5′-GGCATTAAAGCAGCGTATCC-3′ for *tdTomato* positive integration.

Mice were housed in specific pathogen-free animal care facilities using an individually ventilated cage system (Techniplast, Tokyo, Japan) with *ad libitum* access to food and water. The facility conditions were maintained at 23 ± 2 °C, 50 ± 10% relative humidity, and a 14-hour light/10-hour dark cycle. Both male and female mice were used in the analyses unless otherwise specified.

## Antibodies and chemicals

The following antibodies were used for imaging analyses: rabbit anti-Pax6 (MBL International, Tokyo, Japan; Cat# PD022, RRID: AB_1520876), mouse anti-Brn3a (Millipore, Burlington, MA, USA; Cat# MAB1585, RRID: AB_94166), mouse anti-RNA binding protein with multiple splicing (1C12) (RBPMS, Santa Cruz biotechnology, Dallas, TX, USA; Cat# sc-293285, RRID: AB_2910236), rabbit anti-glutamate decarboxylase 65 (GAD65, Sigma-Aldrich, St. Louis, MO; Cat# G4913, RRID: AB_259917), goat anti-choline acetyltransferase (ChAT, Millipore; Cat# AB144P, RRID: AB_2079751), rabbit anti-parvalbumin (E8N2U) XP (PVALB, Cell signaling technology, Danvers, MA, USA; Cat#80561), and Alexa Fluor 488-labeled secondary antibodies (Thermo Fisher, Carlsbad, CA, USA). Cisplatin was obtained from FUJIFILM Wako Pure Chemical Corporation (Osaka, Japan).

## Histochemistry

Mice were anesthetized with a mixture of 0.3 mg/kg medetomidine, 4.0 mg/kg midazolam, and 5.0 mg/kg butorphanol administered via intraperitoneal injection (i.p.). Transcardial perfusion was performed with 4% PFA in 0.1 M phosphate buffer (PB; pH 7.4) [18]. Bilateral eyeballs and optic nerves were dissected, and the eyeballs were enucleated. After post-fixation for 12 h at 4 °C, samples were transferred to 30% sucrose and incubated for 24 h at 4 °C.

The eyeballs were further dissected between the photoreceptor layer (PRL) and the retinal pigment epithelium (RPE) to obtain whole, flat-mounted retinae. Retinae and optic nerves were mounted on glass slides using ProLong Glass Antifade Mountant with NucBlue (Thermo Fisher Scientific) and examined using an LSM900 confocal microscope in camera mode (Carl Zeiss, Oberkochen, Germany). The number of tdTomato-positive cells per mouse (total in the right and left retinae) was quantified.

OTC-embedded samples were used to obtain 10 μm cryostat sections of retinae around the optic disc and optic nerves. For immunostaining, sections were permeabilized with phosphate-buffered saline (PBS) containing 0.3% Triton X-100 (PBS-0.3T) and blocked with 5% fat-free bovine serum albumin (BSA). Sections on glass slides were then incubated with primary antibodies for 2 h at 23 °C in PBS-0.03T containing 3% fat-free BSA, followed by Alexa Fluor 488-labeled secondary antibodies and DAPI nuclear counterstaining for 1 h at 23 °C. Samples were mounted in ProLong Glass Antifade Mountant (Thermo Fisher Scientific) and observed under an LSM900 confocal microscope (40× objective). Orthogonal projection images were generated from each z-stack image (0.48 μm intervals) using ZEN Blue software (Carl Zeiss). The numbers of tdTomato-positive RGCs and ACs (across five cryostat sections per mouse) was analyzed, and the proportions of tdTomato-positive RGCs and ACs were determined. The thicknesses of the retinae from the nerve fiber layer (NFL) to the inner nuclear layer (INL), and from the NFL to the outer nuclear layer (ONL) (Fig. S7), were measured. The percentages of Brn3a-positive or PVALB-positive cells in the ganglion cell layer (GCL) and the number of PVALB-positive cells in the INL (across five images per mouse) were analyzed.

Apoptosis detection was performed using an ApopTag Fluorescein In Situ Apoptosis Detection kit (S7110, Millipore) according to the manufacturer’s protocol. Whole retinae, transcardially fixed with 4% PFA in 0.1 M PB and dissected between the PRL and RPE, were post-fixed with methanol at *−* 20 °C for 20 min on postnatal day 21 (P21). After permeabilization with 20 µg/mL proteinase K for 30 min, whole and flat-mounted retinae were incubated in equilibration buffer for 20 min. Then, terminal deoxynucleotidyl transferase (TdT) and dNTP-digoxigenin were added to the samples and incubated in a humidified chamber at 37 °C for 3 h. The reaction was stopped, and samples were incubated with anti-digoxigenin fluorescein solution and DAPI for 1 h at 23 °C before imaging with an LSM900 confocal microscope (40× objective) using a z-stack imaging mode (0.48 μm intervals). 3D-reconstructed images (lateral projection view) were obtained to identify retinal layers, including the GCL and INL, using ZEN Blue software (Carl Zeiss). The number of TUNEL-positive cells in the GCL and INL (across five images per mouse) was analyzed.

## Reverse transcription (RT)-PCR

To detect *Nox3* mRNA, total RNA from whole retinae—dissected between the PRL and RPE and containing nine retinal layers—was obtained from one mouse in each group (2-month-old (2M) WT, HT *Nox3*-KO, and *Nox3*-KO) using RNA stabilization solution (RNAlater, Thermo Fisher Scientific) and a RNA isolation kit (NucleoSpin RNA, MACHEREY-NAGEL GmbH & Co. KG; Duren, Germany). RT was performed with 2 µg of total RNA using SuperScript III reverse transcriptase (Thermo Fisher Scientific), as previously described [7]. The following primer pair was used: 5′-GTC **ATG** CCG GTG TGC TGG ATT C-3′ and 5′**-CTA** GAA GTT TTC CTT GTT GTA ATA GAA ATG-3′ (predicted product size: 1710 bp) for PCR with 35 cycles.

## ERG

The ERG test measures the electrical activity of the retina in response to a light stimulus. It is an objective measure of retinal function recorded noninvasively under physiological conditions. ERGs are recorded using an electrode embedded within a corneal contact lens. These electrodes allow the electrical activity generated by the retina to be recorded at the corneal surface. The STR-wave reflects RGC function, the a-wave reflects photoreceptor cell function, and the b-wave reflects bipolar cell function [19, 20]. Furthermore, ACs may also affect the b-wave [19–21].

ERG recordings were performed as previously described [22, 23]. Briefly, mice were dark-adapted for 16 h before anesthesia with ketamine (100 mg/kg, i.p.) and xylazine (9 mg/kg, i.p.). Pupil dilation was induced using 1% tropicamide and 2.5% phenylephrine (Santen, Osaka, Japan). Flash ERG was recorded using a gold ring electrode (Mayo, Aichi, Japan) placed on the cornea, with a reference electrode (Nihon Kohden, Tokyo, Japan) inserted into the tongue and a neutral electrode (Nihon Kohden, Tokyo, Japan) placed subcutaneously near the tail. Flash ERG was recorded for both eyes under white fluorescent stimuli at intensities from − 1.4 to 0.6 log cd·s/m^2^ for a-waves and b-waves, and − 6.0 to −4.5 log cd·s/m^2^ for STR-waves. The a-wave amplitude was measured from the baseline to the trough of the a-wave, the b-wave amplitude from the trough of the a-wave to the peak of the b-wave, and the STR-wave amplitude from the baseline to the peak of the STR-wave. Amplitudes of a-, b-, and STR-waves were calculated by averaging the data from both eyes.

### Cisplatin treatment

The cisplatin treatment protocol is shown in the figure. Briefly, cisplatin was dissolved in saline and administered intraperitoneally at a dose of 5 mg/kg for 6 consecutive days to 1M, 2M, and 6M *Nox3-Cre*^*+/−*^;*tdTomato*^*+/+*^ (HT *Nox3*-KO) and *Nox3-Cre*^*+/+*^;*tdTomato*^*+/+*^ (*Nox3*-KO) mice. On day 7, mice were transcardially fixed after intraperitoneal anesthesia with medetomidine (0.3 mg/kg), midazolam (4.0 mg/kg), and butorphanol (5.0 mg/kg). Whole and flat-mounted retinae were used in the analyses. The experimental endpoint was defined as ≥ 25% loss in body weight by day 4, based on daily body weight measurements.

## Experimental design and statistical analysis

All data are presented as mean ± SEM. Comparisons between two groups were conducted using an unpaired, two-tailed Student’s t-test, while comparisons among multiple groups were analyzed using one-way or two-way ANOVA, followed by Tukey’s post hoc test for pairwise group differences. Statistical analyses were performed using Prism 7.0 software (GraphPad Software, La Jolla, CA, USA). Significant differences are indicated as *P < 0.05, **P < 0.01, ***P < 0.001, and ****P < 0.0001. Complete statistical details, including exact *n* and *p*-values, and the statistical tests performed, are provided in figures and/or figure captions.

## Results

### Expression of Nox3 in Pax6-positive RGCs and ACs in the retina

Previously, we generated *Nox3-Cre* KI mice in which *Cre* was knocked into the *ATG* site of *Nox3* (Fig. S1) [7]. To examine Nox3-expressing cells, *Nox3-Cre* KI mice were intercrossed with *CAG-STOP*^*flox*^*-tdTomato* reporter mice, and the offspring are referred to as *Nox3-Cre*^*+/−*^;*tdTomato*^+/*−*^, in which tdTomato was expressed in cells under a functionally active *Nox3* promoter-driven Cre. *Nox3-Cre*^*+/−*^;*tdTomato*^+/*−*^ mice were further crossed with *Nox3-Cre*^*+/−*^;*tdTomato*^+/*−*^ to obtain *Nox3-Cre*^*−/−*^;*tdTomato*^*+/+*^, *Nox3-Cre*^*+/−*^;*tdTomato*^*+/+*^, and *Nox3-Cre*^*+/+*^;*tdTomato*^*+/+*^ populations (Fig. S1) [7]. Regarding *Nox3*, the first one is WT without tdTomato fluorescence, while in the latter two, exon 1 of *Nox3* was replaced with *Cre* at one or both alleles, respectively, resulting in heterozygous *Nox3*-KO (HT *Nox3*-KO) and homozygous *Nox3*-KO (*Nox3*-KO) mice with tdTomato fluorescence (Fig. S1). Additionally, we previously reported that Nox3 mRNA expression in the inner ear was at nearly the same levels in WT and HT *Nox3*-KO mice, suggesting that HT *Nox3*-KO mice can likely be used as WT mice [7].

**Fig. 1:**
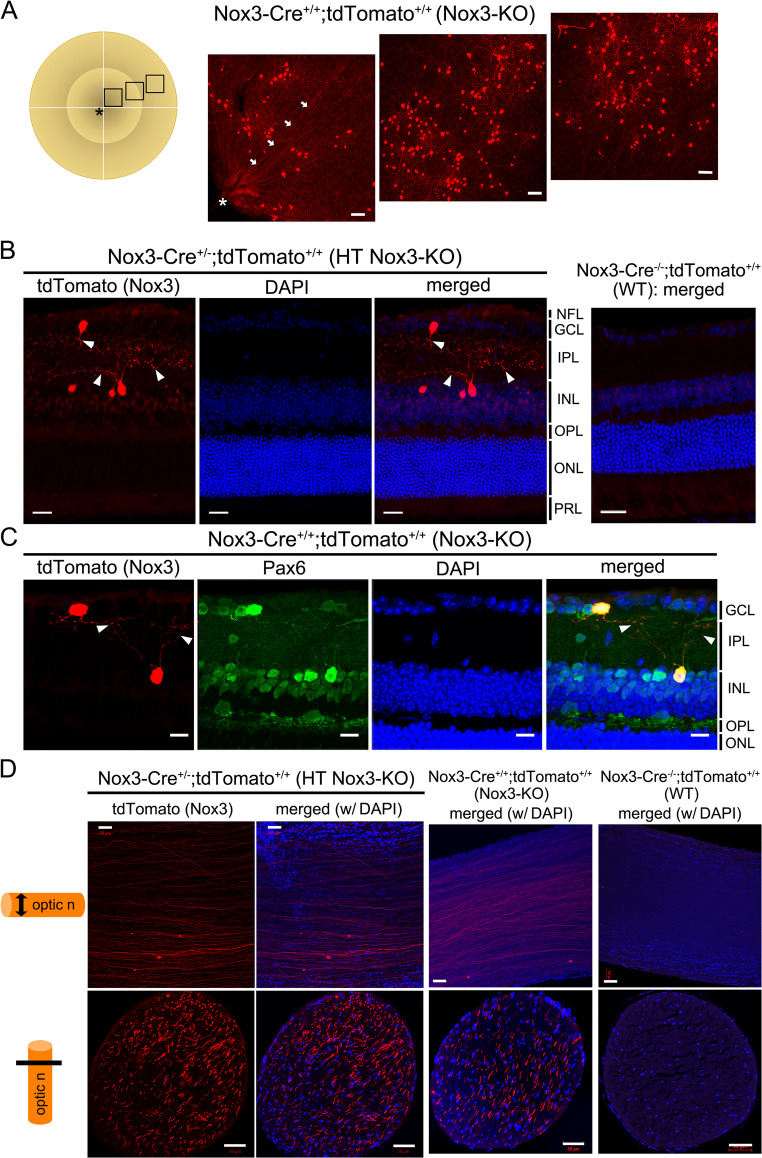
Nox3 expression in Pax6-positive retinal ganglion cells (RGCs) and amacrine cells (ACs) as well as optic nerves. Whole-mount (**A**) and cryostat sections (**B** and **C**) of retinae and whole-mount (**D**, upper) and cryostat sections (**D**, lower) of optic nerves were prepared from *Nox3-Cre*^-/-^; *tdTomato*^+/+^ (WT), *Nox3-Cre*^+/-^; *tdTomato*^+/+^ (heterozygous [HT] *Nox3-KO*), and *Nox3-Cre*^+/+^; *tdTomato*^+/+^ (*Nox3*-KO) mice. (**A**) tdTomato-positive cells in 2-month-old (2M) *Nox3*-KO retinae. The squares in the retinal illustration indicate the imaging sites. Asterisks and arrows denote the optic disc and an optic nerve fiber, respectively. Scale bars: 50 μm. (**B**) Cryostat sections of 6M HT *Nox3*-KO and WT retinae stained with DAPI. tdTomato-positive cells with neurites (arrowheads) are present in the ganglion cell layer (GCL) and inner nuclear layer (INL). NFL, nerve fiber layer; IPL, inner plexiform layer; OPL, outer plexiform layer; ONL, outer nuclear layer; PRL, photoreceptor layer. Scale bars: 25 μm. (**C**) Cryostat sections of 4M *Nox3*-KO retinae stained with Pax6 and DAPI counterstaining. tdTomato-positive neurites with punctuated signals (arrowheads) from RGCs appear to form synapses between the ACs (**B** and **C**). Scale bars: 10 μm. (**D**) Whole-mount (upper) and cryostat sections (lower) of 12M HT* Nox3*-KO, Nox3-KO, and WT optic nerves stained with DAPI. Scale bars: 50 μm

Expression of full-length *Nox3* mRNA (from start to stop codons) in WT and HT *Nox3*-KO, but not *Nox3*-KO retinae, was confirmed by RT-PCR (Fig. S2). Whole-mount retinae were prepared from *Nox3-Cre*^*+/+*^;*tdTomato*^*+/+*^ (*Nox3*-KO) mice to examine Nox3 expression in the retina. tdTomato fluorescence-positive cells and fiber structures were observed in the retina (Fig. 1A), suggesting that these cells were RGCs and their nerve fibers. The tdTomato-positive cells were not evenly distributed; instead, they were localized in punctate accumulations in the retina (Fig. 1A, Fig. S3). Next, cryostat sections showing nine retinal layers (from the inner limiting membrane to the PRL) were prepared. tdTomato-positive cells were observed in the GCL and INL (Fig. 1B). Additionally, these two cell types appeared interconnected by their fibers with dot-like formations (Fig. 1B, C). Immunostaining was performed using various antibodies to determine the cell types of tdTomato-positive cells [24, 25]. The tdTomato-positive cells were Pax6-positive (Fig. 1C), a marker for mature RGCs in the GCL [26] and pan-ACs in the INL [24]. Furthermore, tdTomato-positive cells in the GCL were positive for Brn3a and RBPMS, markers for RGCs (Fig. S4). tdTomato-positive cells in the INL were positive for GAD65, a marker for GABAergic ACs, but negative for ChAT, a marker for cholinergic ACs (Fig. S4). Additionally, tdTomato-positive cells in the INL were primarily negative for PVALB, a marker for RGCs and ACs [27, 28] (Fig. S4). tdTomato fluorescence was also detected in both whole-mount and cryostat sections of the optic nerve (Fig. 1D). Taken together, tdTomato-positive cells are RGCs—excitatory and third neurons in the visual pathway—and at least GABAergic ACs—inhibitory interneurons regulating RGC and bipolar cell functions [29].

**Fig. 2:**
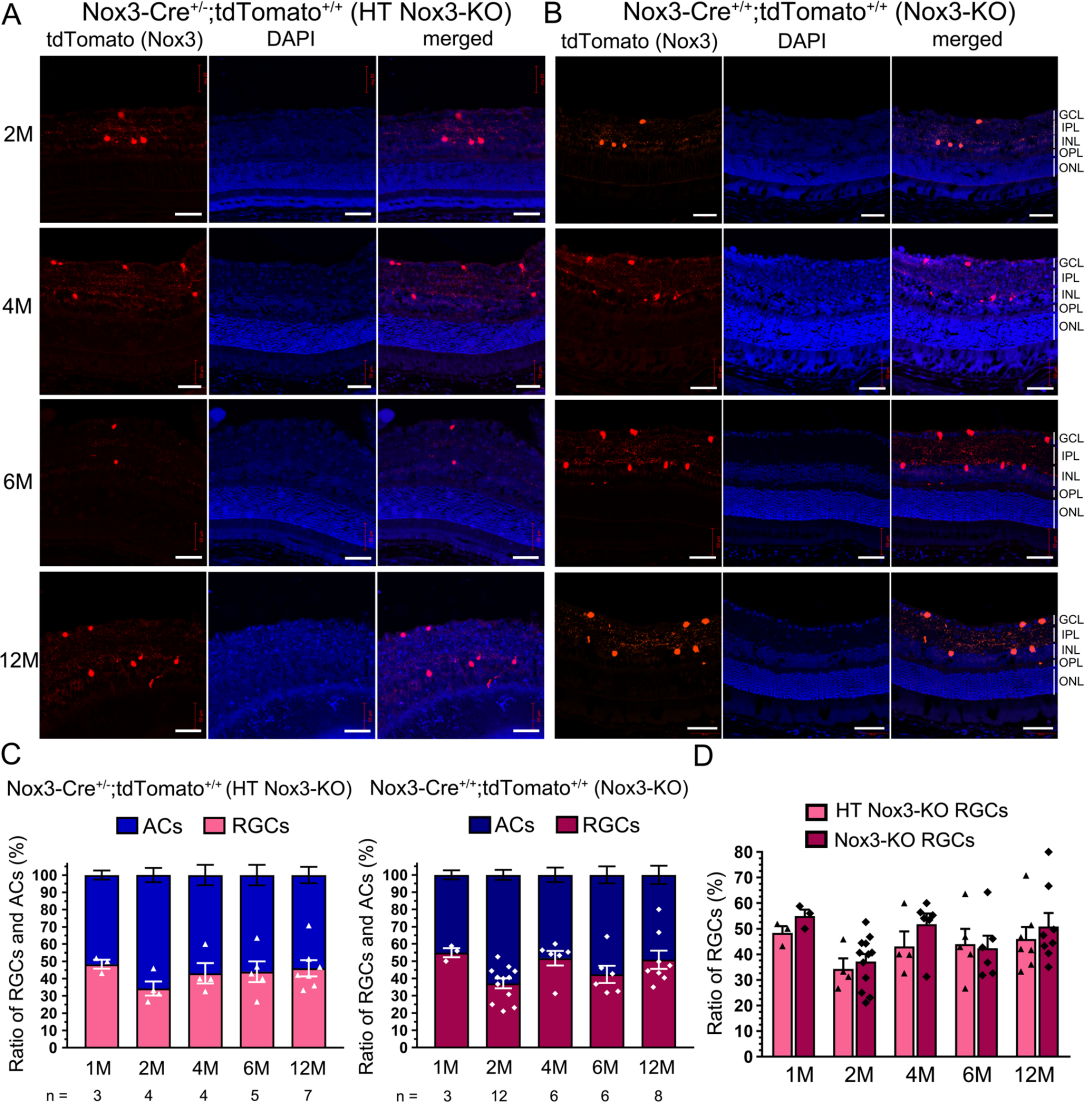
Comparable time course of tdTomato-positive retinal ganglion cells (RGCs) and amacrine cells (ACs) ratios between HT *Nox3*-KO and *Nox3*-KO mice. Cryostat sections of retinae were prepared from 1-month-old (1M), 2M, 4M, 6M, and 12M *Nox3-Cre*^+/-^; *tdTomato*^+/+^ (heterozygous [HT] *Nox3*-KO), *Nox3-Cre*^+/+^; *tdTomato*^+/+^ (*Nox3*-KO), and *Nox3-Cre*^-/-^; *tdTomato*^+/+^ (WT) mice. (**A** and **B**) Representative images of HT *Nox3*-KO (**A**) and *Nox3*-KO (**B**) mice. Scale bars: 50 μm. Representative images of WT mice are available in Fig. S5. GCL, ganglion cell layer; IPL, inner plexiform layer; INL, inner nuclear layer; OPL, outer plexiform layer; ONL, outer nuclear layer. (**C**) Percentages of RGCs and ACs are plotted in HT *Nox3*-KO and *Nox3*-KO mice. No significant differences were detected using two-way ANOVA with Tukey’s post hoc test. The number of mice analyzed (n) is shown in the graph. (**D**) RGC percentages at each age in HT *Nox3*-KO and *Nox3*-KO mice are presented in graphical form. No significant differences were detected using Student’s t-test

Although displaced ACs, which are located in the GCL, occupy 40–59% of neurons in the rodent GCL [30–32], and displaced RGCs in the INL constitute about 2% of the total RGC population [32, 33], cryostat sections of retinae from 1M to 12M mice were examined to analyze the ratio of tdTomato-positive cells in the GCL (as RGCs) and in the INL (as ACs) (Fig. 2A, B; Fig. S5), In HT *Nox3*-KO mice, the ratio of ranged from 34.3 ± 4.1% to 48.4 ± 2.6%, with the lowest at 2M and the highest at 1M (Fig. 2C). In *Nox3*-KO mice, the ratio ranged from 37.2 ± 2.9% to 54.9 ± 2.6%, with the lowest at 2M and the highest at 1M (Fig. 2C). No significant difference in the ratio was observed between HT *Nox3*-KO and *Nox3*-KO mice (Fig. 2C, D). Additionally, the percentage of Brn3a-positive or PVALB-positive cells in the GCL and the number of PVALB-positive cells in the INL were not significantly different between WT and *Nox3*-KO mice at P10, P21, or 4M (Fig. S6).

**Fig. 3:**
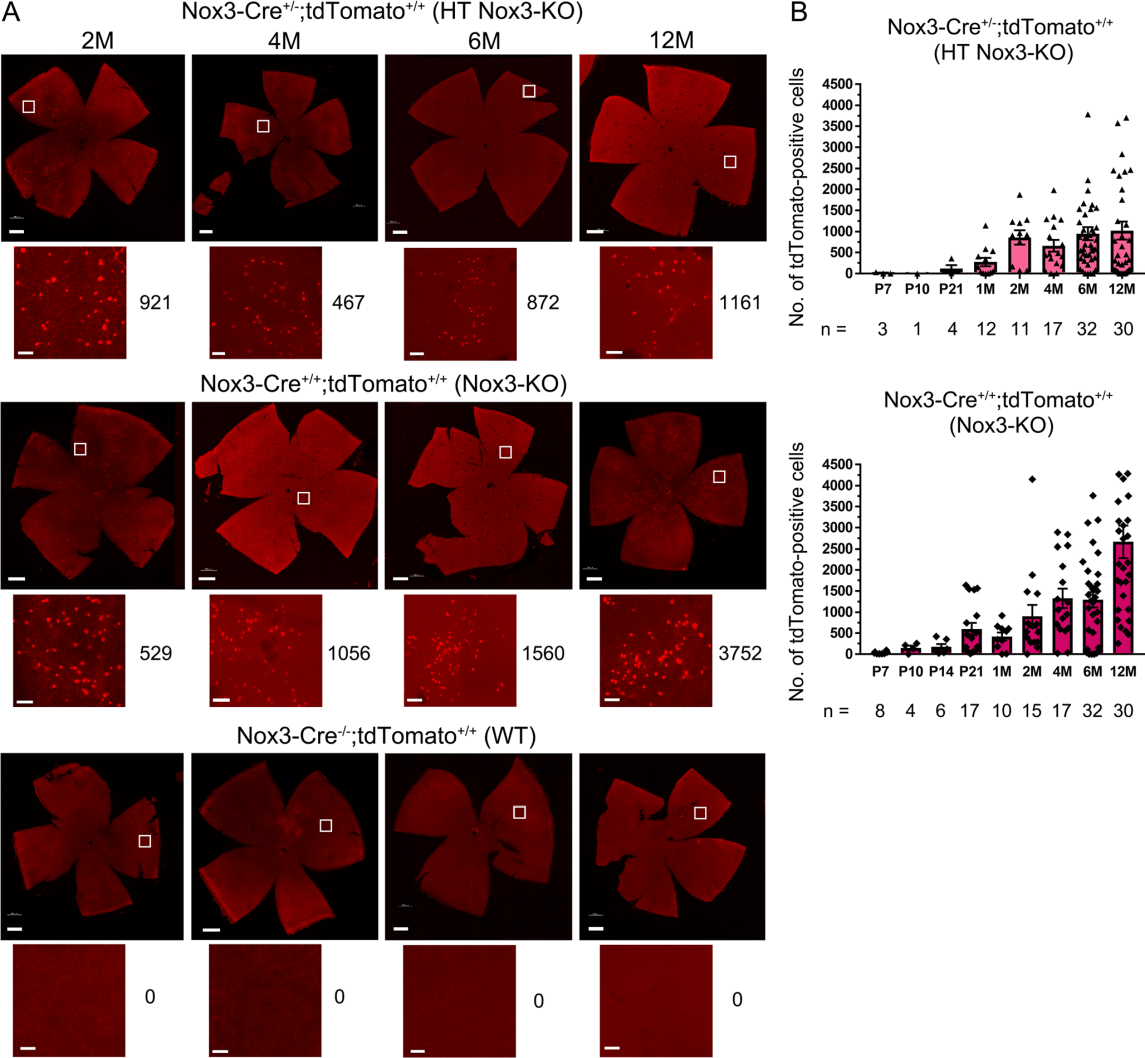
Distinct time courses of tdTomato-positive cells in HT* Nox3*-KO and *Nox3*-KO mice. Whole-mount retinae were prepared from postnatal day 7 (P7), P10, P14, P21, 1-month-old (1M), 2M, 4M, 6M, and 12M *Nox3-Cre*^+/-^; *tdTomato*^+/+^ (heterozygous [HT] *Nox3*-KO), *Nox3-Cre*^+/+^; *tdTomato*^+/+^ (*Nox3*-KO), and *Nox3-Cre*^-/-^; *tdTomato*^+/+^ (WT) mice. (**A**) Representative images of the retinae (upper panels in each genotype, scale bars: 500 μm) and magnified images (lower panels in each genotype, scale bars: 50 μm) indicated by squares. The number of tdTomato-positive cells per mouse (total in the right and left retinae) is shown on the right side of the magnified image. (**B**) tdTomato-positive cell counts per mouse were quantified and plotted in HT *Nox3*-KO and *Nox3*-KO mice. The number of mice analyzed (n) is indicated in the graph

*Nox3*-KO mice showed no abnormal morphological development of the retina compared to *Nox3-Cre*^*−/−*^;*tdTomato*^*+/+*^ (WT) and *Nox3-Cre*^*+/−*^;*tdTomato*^*+/+*^ (HT *Nox3*-KO) retinae (Figs. 1A and C and 3A). The thicknesses of retinae from the NFL to INL, where tdTomato-positive cells were present, and from the NFL to the ONL were not significantly different between WT and *Nox3*-KO mice at P10, P21, or 2M (Fig. S7).

### Time course of Nox3 expression: HT Nox3-KO vs. Nox3-KO

The time course of Nox3 expression was analyzed by quantifying tdTomato-positive cells in whole-mount retinae from P7 to 12M. tdTomato-positive cells increased by 2M and then plateaued in HT *Nox3*-KO mice (Fig. 3A, B). In sharp contrast, a time-dependent increase was observed in *Nox3*-KO mice (Fig. 3A, B). Furthermore, apoptosis was examined using the TUNEL assay at P21, when a difference in the number of tdTomato-positive cells was observed between HT *Nox3*-KO and *Nox3*-KO mice (Fig. 3B) and mouse retinal development is complete [34]. No significant difference in TUNEL-positive cells in the GCL and INL was observed between WT and *Nox3*-KO mice (Fig. S8).

### Reduced ERG response in Nox3-KO mice

**Fig. 4:**
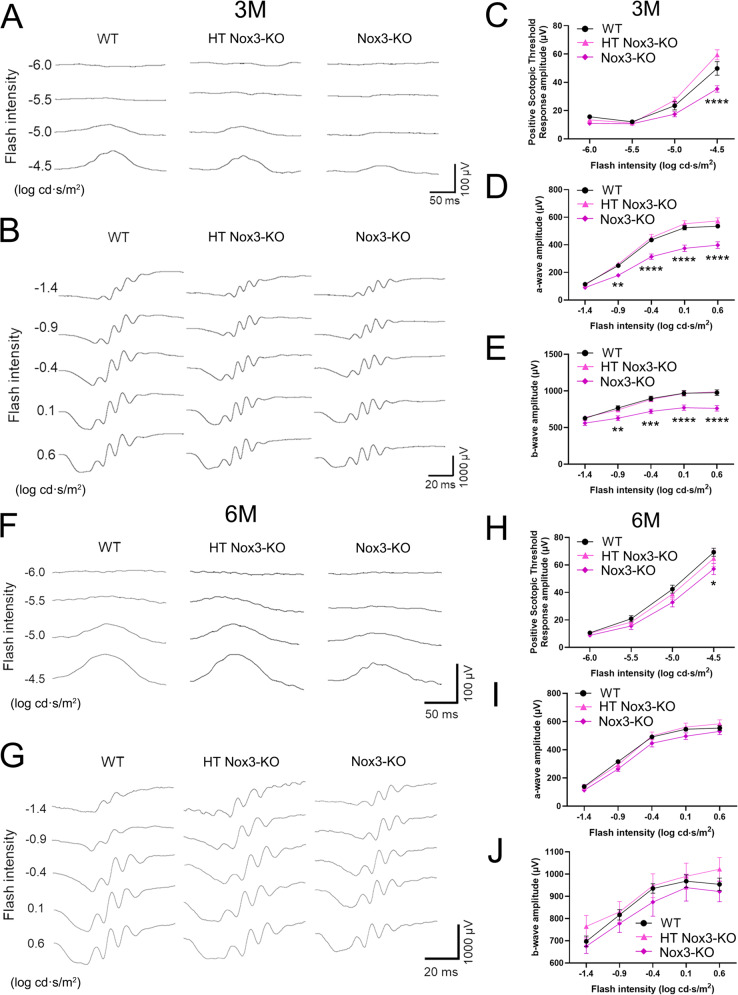
Reduced electroretinogram (ERG) response in *Nox3*-KO mice. ERG was recorded in 3-month-old (3M) and 6M *Nox3-Cre*^-/-^; *tdTomato*^+/+^ (wild-type [WT]), *Nox3-Cre*^+/-^; *tdTomato*^+/+^ (heterozygous [HT] *Nox3*-KO), *Nox3-Cre*^+/+^; *tdTomato*^+/+^(*Nox3*-KO) mice. Significant differences (WT vs.* Nox3*-KO) were detected using two-way ANOVA with Tukey’s post hoc test. (**A** and **B**) Representative STR wave (**A**) and a-wave and b-wave (**B**) recordings in 3M WT, HT *Nox3*-KO, and *Nox3*- KO mice. (**C**) Quantification of STR wave amplitude at 3M. n = 12. WT vs. Nox3-KO: ****P < 0.0001. (**D**) Quantification of a-wave amplitude at 3M. n = 12. WT vs. *Nox3*-KO: -0.9 log cd·s/m²; **P = 0.0084, -0.4 log cd·s/m²; ****P < 0.0001, 0.1 log cd·s/m²; ****P < 0.0001, 0.6 log cd·s/m²; ****P < 0.0001. (**E**) Quantification of b-wave amplitude at 3M. n = 12. WT vs. *Nox3*-KO: -0.9 log cd·s/m²; **P = 0.0064, -0.4 log cd·s/m²; ***P = 0.0004, 0.1 log cd·s/m²; ****P < 0.0001, 0.6 log cd·s/m²; ****P < 0.0001. (**F** and **G**) Representative STR wave (**F**), a-wave, and b-wave (**G**) recordings in 6M WT, HT *Nox3*-KO, and *Nox3*-KO mice. (**H**) Quantification of STR wave amplitude at 6M. n = 13 (WT), 10 (HT *Nox3*-KO), and 7 (*Nox3*-KO) mice. *P= 0.0240. (**I**, **J**) Quantification of a-wave amplitude (**I**) and b-wave (**J**) amplitudes at 6M. n = 13 (WT), 10 (HT *Nox3*- KO), and 7 (*Nox3*-KO) mice.= 0.0240. (**I**, **J**) Quantification of a-wave amplitude (**I**) and b-wave (**J**) amplitudes at 6M. n = 13 (WT), 10 (HT *Nox3*-KO), and 7 (*Nox3*-KO) mice

To investigate the role of Nox3 in retinal function, ERG was performed on *Nox3-Cre*^*−/−*^;*tdTomato*^*+/+*^ (WT), *Nox3-Cre*^*+/−*^;*tdTomato*^*+/+*^ (HT *Nox3*-KO), and *Nox3-Cre*^*+/+*^;*tdTomato*^*+/+*^ (*Nox3*-KO) mice. *Nox3*-KO mice at 3M showed a significant reduction in the STR-wave, reflecting RGC function [35, 36]; as well as in the a-wave and b-wave, reflecting bipolar cell and AC function [19–21], compared to WT mice (Fig. 4A–E). Although less pronounced than that at 3M, 6M *Nox3*-KO mice showed a significant reduction in the STR waves at −4.5 flash intensity compared to WT mice (Fig. 4F, H). No significant differences were observed in the a- and b-waves among 6M WT, HT *Nox3*-KO, and *Nox3*-KO mice (Fig. 4G, I, J). These findings suggest that Nox3-derived ROS play a more crucial role in STR waves than in a- and b-waves.

### Nox3-KO mice resist cisplatin-induced loss of tdTomato-positive cells in the retina

**Fig. 5:**
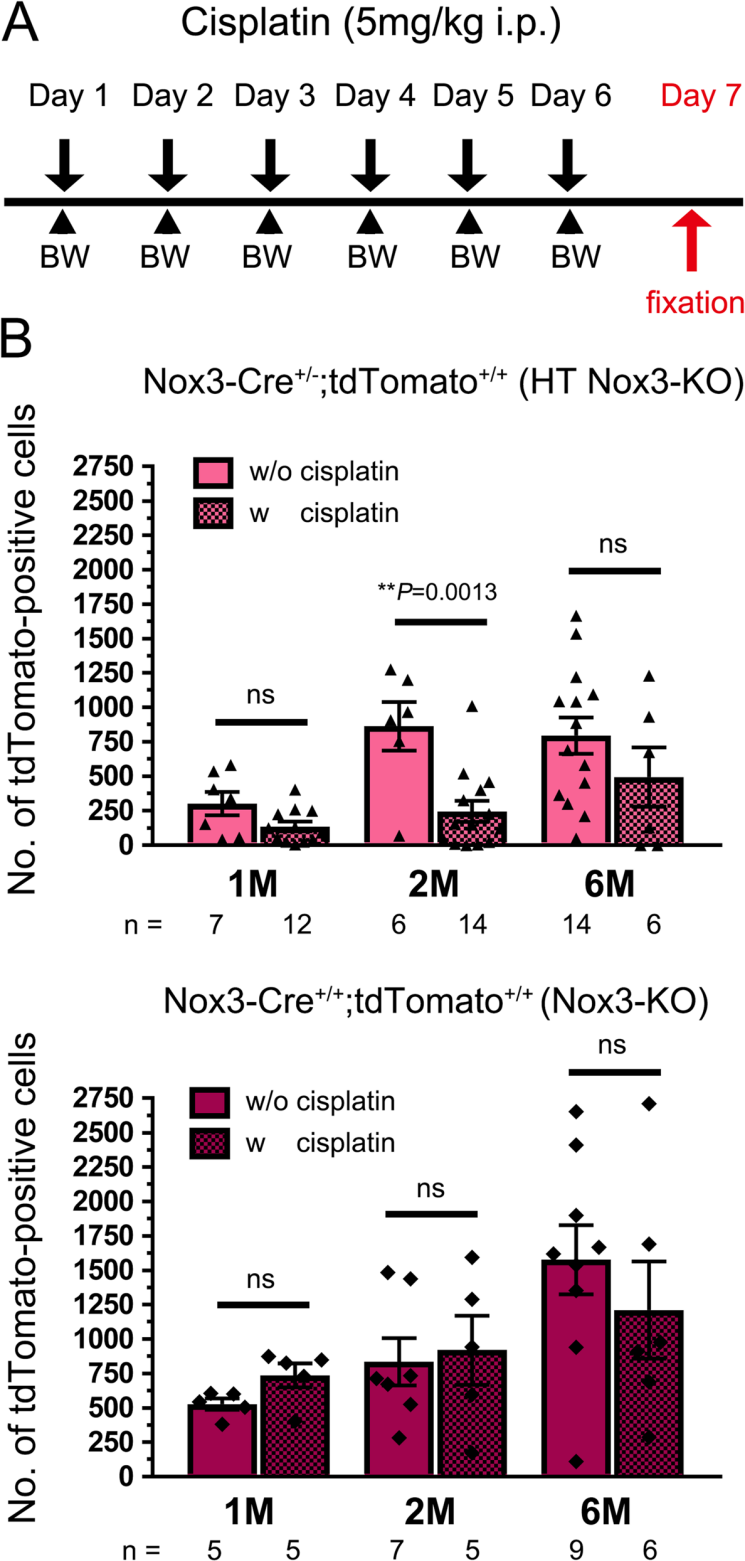
Ameliorated tdTomato-positive cell loss in *Nox3*-KO retinae after cisplatin treatment. One-month-old (1M), 2M, and 6M *Nox3-Cre*^+/-^; *tdTomato*^+/+^ (heterozygous [HT] *Nox3*-KO) and *Nox3-Cre*^+/+^; *tdTomato*^+/+^ (*Nox3*-KO) mice were treated with cisplatin following the protocol outlined in **A**. After fixation, whole-mount retinae were prepared, and tdTomato-positive cells were quantified and plotted (**B**). Significant differences were detected using the Student’s t-test in 2M HT *Nox3*-KO mice (**P = 0.0013), but not in 1M (*P* = 0.0506), 6M (*P* = 0.2382) HT *Nox3*-KO mice, or *Nox3*-KO mice. The number of mice analyzed is shown in the graphs. ns, not significant

The effects of cisplatin, known to cause blurred vision and altered color perception [37] due to retinal edema and retinal toxicity leading to RGC loss, papilledema, and optic neuritis [38–40], were examined using *Nox3-Cre*^*+/−*^;*tdTomato*^*+/+*^ (HT *Nox3*-KO) mice, as a substitute for WT mice [7], and *Nox3-Cre*^*+/+*^;*tdTomato*^*+/+*^ (*Nox3*-KO) mice, following the protocol outlined in Fig. 5A. In HT *Nox3*-KO mice, cisplatin treatment significantly reduced tdTomato-positive cells in the retinae compared to untreated retinae at 2M, but not at 1M or 6M (*P* = 0.0013 at 2M, *P* = 0.0506 at 1M, *P* = 0.2382 at 6M; Fig. 5B). In sharp contrast, no significant difference was observed between cisplatin-treated and untreated retinae in *Nox3*-KO mice, at 1M, 2M, or 6M (Fig. 5B). These findings suggest that Nox3-derived ROS mediate cisplatin-induced death of tdTomato-positive cells.

## Discussion

Aside from studies focusing on conjunctival fibroblasts in rabbits and corneal stromal fibroblasts in humans, Nox3 has not been extensively investigated in the eye [5]. While Nox3 mRNA has been detected in rabbit conjunctival fibroblasts [41], it was not found in human corneal stromal fibroblasts [42]. In this study, using *Nox3-Cre;tdTomato*^*+/+*^ mice, we identified Nox3 expression in mouse RGCs and GABAergic ACs, which regulate RGC and bipolar cell functions through synaptic interactions [43, 44]. Using single-cell RNA sequencing, which allows a better understanding of the link between anatomy, gene expression, and function, in addition to morphological and physiological methods, 40–47 subtypes of RGCs [34, 45–47] and 63–67 subtypes of ACs [47, 48] were molecularly and genetically identified in mice. In mouse ACs, 67–70% are GABAergic followed by 22.5–25% of glycinergic ACs [47, 48]. Although we did not examine colocalization of tdTomato fluorescence with markers other than GABAergic and cholinergic ACs, the single-cell atlas of the mouse retina, established by the Chen Lab using their own data and public datasets (CELLxGENE, https://rchenlab.github.io/resources/mouse-atlas.html) and their report [47], showed expression of Nox3 in RGCs, GABAergic ACs, and glycinergic ACs, but not in non-GABAergic/non-glycinergic (nGnG) ACs or dual positive ACs.

Previously, we reported that HT *Nox3*-KO mice likely exhibit ROS production capabilities comparable to WT mice [7]. In the present study, tdTomato-positive cells increased by 2M and plateaued by 12M in HT *Nox3*-KO mice, whereas in *Nox3*-KO mice, tdTomato-positive cells increased by 12M. These findings suggest that Nox3-derived ROS may exert toxic effects on RGCs and ACs as early as 4M. Although we did not determine the percentage of displaced RGCs and ACs or whether RGCs or ACs were more susceptible to cisplatin-induced cell death, the absence of significant differences in their proportions in aging (1M–12M, see Fig. 2C) suggests that both RGCs and ACs may have comparable sensitivity to Nox3-derived ROS.

It is intriguing that tdTomato-positive cells were not evenly distributed but appeared sporadically in the retina. While the underlying mechanism of Nox3 expression and distribution remains unclear, this irregularity may be attributed to the transcriptional characteristics of Nox3, which is induced by ROS [5, 49] and pro-inflammatory cytokines such as TNF-α, IL-1β, and INF-γ [12, 13, 50]. This hypothesis—that focal inflammation induces Nox3 expression—is supported by findings that etanercept, a TNF-α inhibitor, downregulates *Nox3* mRNA expression [50]. However, excessive ROS production and severe inflammation are cytotoxic. In the inner ear, cisplatin has been reported to induce a 2.5-fold increase in *Nox3* mRNA expression [51]. We previously demonstrated that cisplatin induces Nox3 expression in cochlear hair cells and supporting cells; however, Nox3-expressing outer hair cells, which are more vulnerable to ROS than inner hair cells and supporting cells, undergo apoptosis [7]. In the present study, cisplatin treatment did not significantly increase the number of tdTomato-positive cells (RGCs and ACs) in *Nox3*-KO mice, but reduced in HT *Nox3*-KO mice, suggesting that Nox3 transcription may not be activated by cisplatin in the retina as much as in the cochlea, or that cisplatin-induced death of tdTomato-positive cells counteracted any increase in Nox3 expression. The former speculation may be supported by the observation that cisplatin side effects in the retina/vision are less frequent than in cochlea/hearing [52, 53], and that cisplatin reportedly enhances ROS production from Nox3 without *Nox3* transcriptional activation [10]. Further studies are required to determine the Nox3 effects via cisplatin on the retina, as well as the redox signaling status in *Nox3*-KO mice compared with WT mice.

In this study, we identified the effects of Nox3 on ERG (STR-, a-, and b-waves). Although no significant differences were observed between WT and *Nox3*-KO mice, PVALB-positive cells in the GCL (P21 and 4M) and INL (4M) tended to decrease in *Nox3*-KO mice after retinal development [34]. PVALB is a Ca^2+^-binding protein, expressed in RGCs and ACs, and it functions in Ca^2+^ buffering [28]. PVALB reportedly played roles in antioxidant effects [54], neuroprotection against glutamate toxicity [28], and determining cell survival [27]. In transcriptome analysis using cochleae from *p22*^*phox*^-KO mice—considered pan-Nox-KO mice (targeting Nox1, Nox2, Nox3, and Nox4)—the downregulated genes were primarily associated with Ca^2+^ and glutamate signaling [55]. Taken together, the decrease in STR- and b-waves in *Nox3*-KO mice may be fundamentally due to RGC and AC dysfunction caused by the lack of Nox3-derived ROS, which are released into the extracellular space from the plasma membrane [9] and are associated with Ca^2+^ and glutamate signaling. Furthermore, since some of the tdTomato-positive RGCs were also positive for PVALB, mild RGC loss resulting from RGC dysfunction may lead to decreased STR-waves in *Nox3*-KO mice.

ERG effects on *Nox3*-KO mice were more pronounced at 3M (STR-, a-, and b-waves) than at 6M (restricted to STR-waves). Similarly, cisplatin-induced effects on HT *Nox3*-KO mice were more significant in younger mice (1M and 2M) than in older mice (6M). These findings suggest that Nox3-derived ROS exert a more significant functional effect in younger mice (1–3M) than in older mice (6M). Consistent with these data, we previously reported that cisplatin effects in the inner ear are higher in 1M and 2M mice than in 6M mice [7]. Although age-dependent retinal toxicity of cisplatin has not been reported in mice or humans, age-dependent ototoxicity has been reported in humans [56]. Taken together, aged mice (6M HT *Nox3*-KO mice in the present study) [57] may develop resistance to Nox3-derived ROS. Indeed, some ROS-scavenging molecules, such as superoxide dismutase and glutathione hydrolase 7, have been reported to increase in mice up to 12–14M of age [58]. Additionally, as the number of tdTomato-positive cells plateaued at 2M in HT *Nox3*-KO mice, the functions and effects of Nox3-derived ROS may change between 2M and 6M. Moreover, since tdTomato-positive cells increased significantly between 6M and 12M, the functions of Nox3-derived ROS may further change during this period.

Regarding Nox3’s roles during retinal development, Nox3 is unlikely to be involved in retinal morphology because of the normal shape and thickness of *Nox3*-KO retinae. This speculation is supported by the result that the number of tdTomato-positive cells in HT *Nox3*-KO mice was low until P21, when mouse retinal development is complete [34]. The accelerated increase in the number of tdTomato-positive cells at P21 observed in *Nox3*-KO mice may be explained by eye opening, which occurs between P12 and P14 [34]. However, to our knowledge, there are no reports showing Nox3 induction by light. Thus, Nox3 is likely involved in retinal function, but not morphology, as detected by ERG, similarly to its role in cochlear function detected by audiometry [7]. Further studies are needed to elucidate the functional difference of Nox3 in young and old mice, as well as Nox3’s response to environmental factors, including light. Also, the mechanism by which Nox3 expression in RGCs and ACs affects the a-wave (which reflects the functions of photoreceptor cells) remains unknown. However, the effect on a-wave may be explained by multiple synaptic network regulations in the visual pathway (photoreceptor cells–bipolar cells–RGCs) modulated by ACs and horizontal cells [59, 60].

Glutamate excitotoxicity and oxidative stress are major pathological mechanisms in retinal disorders [61], and Nox-derived ROS have been implicated in diabetic retinopathy via microvascular dysfunction [62]. In the retina, Nox1, Nox2, and Nox4 have been detected in RGCs, microglia, and pericytes. Nox1 and Nox4 (but not Nox2) are expressed in glial cells, while Nox2 and Nox4 (but not Nox1) are expressed in endothelial cells [63–65]. Additionally, Nox-derived ROS have been linked to glaucoma [65, 66]. However, the role of Nox3 in retinal disorders remains entirely unknown [5]. Further research is required to elucidate Nox3 function in retinal disorders.

## Supplementary Information

Below is the link to the electronic supplementary material.

Supplementary figure 1 *Nox3-Cre;tdTomato* mice for detection of Nox3 expression. Illustration depicting the genetic construction of *Nox3* mutant mice (*Nox3-Cre* knock-in [KI]), in which *Cre recombinase *with a* poly(A)* sequence was inserted into the *ATG* site of exon 1 of *Nox3*. *Nox3-Cre* KI mice were crossed with *CAG-stop*^flox^-*tdTomato* mice to obtain *Nox3-Cre*^-/-^;tdTomato^+/+^ (WT/control), *Nox3-Cre*^+/-^;tdTomato^+/+^ (heterozygous [HT] *Nox3 *knockout [KO]), and *Nox3-Cre*^+/+^;tdTomato^+/+^ (*Nox3*-KO) lines. (PNG 979 KB)

High Resolution Image (TIF 1.66 MB)

Supplementary figure 2 Detection of *Nox3* mRNA in WT and HT *Nox3*-KO, but not in *Nox3*-KO retinae. Reverse transcription was performed using 2 mg of total RNA from 2-month-old *Nox3-Cre*^-/-^;tdTomato^+/+^ (WT), *Nox3-Cre*^+/-^;tdTomato^+/+^ (HT *Nox3*-KO), and *Nox3-Cre*^+/+^;tdTomato^+/+^ (*Nox3*-KO) retinae. PCR using the *Nox3-*specific primer pair yields 1710 bp bands (including both start and stop codons, indicated by an arrowhead) in WT and HT *Nox3*-KO, but not in *Nox3*-KO retinae. (PNG 270 KB)

High Resolution Image (TIF 4.55 MB)

Supplementary figure 3 Punctate accumulations of tdTomato-positive cells in the retina.Whole-mount retinae (scale bars: 500 μm) were prepared from 2-month-old (2M) *Nox3-Cre*^+/-^;tdTomato^+/+^(HT *Nox3*-KO) and 2M and 12M* Nox3-Cre*^+/+^;tdTomato^+/+^ (*Nox3*-KO) mice. Magnified images (scale bars: 100 μm) indicated by squares are shown. tdTomato-positive cells are not evenly distributed but are clustered in regions in the retina. (PNG 1.29 MB)

High Resolution Image (TIF 4.78 MB)

Supplementary figure 4 Immuno histological identification of tdTomato-positive cells in the GCL and INL. Cryostat sections of retinae were prepared from *Nox3-Cre*^+/-^;tdTomato^+/+^ (HT *Nox3*-KO) mice for immunostaining with DAPI counterstaining. tdTomato-positive cells in the ganglion cell layer (GCL) are positive for Brn3a (**A**, arrowheads) and RBPMS (**B**, arrowheads), indicating that they are retinal ganglion cells (RGCs). tdTomato-positive cells that are negative for Brn3a (**A**, yellow arrows) may be displaced amacrine cells (ACs). tdTomato-positive cells in the inner nuclear layer (INL) were positive for GAD65 (**C**, arrowheads), but not for ChAT (**D**, yellow arrows), indicating that they are GABAergic ACs. A tdTomato-positive cell that is negative for GAD65 (**C**, yellow arrows) is detected. tdTomato-positive ACs are primarily negative for PVALB (**E**), but some cells in the GCL that are positive for both tdTomato and PVALB (**E**, arrows) were detected. Scale bars: 20 μm. (PNG 2.07 MB)

High Resolution Image (TIF 8.53 MB)

Supplementary figure 5 No tdTomato-positive retinal ganglion cells (RGCs) or amacrine cells (ACs) in WT mice.Cryostat sections of 2-month-old (2M), 4M, 6M, and 12M *Nox3-Cre*^-/-^;tdTomato^+/+^ (WT) retinae were stained with DAPI. No tdTomato-positive cells exist in the ganglion cell layer (GCL) or inner nuclear layer (INL) at all ages. IPL, inner plexiform layer; OPL, outer plexiform layer; ONL, outer nuclear layer. Scale bars: 50 μm. (PNG 1.41 MB)

High Resolution Image (TIF 5.64 MB)

Supplementary figure 6 Developmental changes in Brn3a-positive and PVALB-positive cells in WT and *Nox3*-KO mice. Cryostat sections of postnatal day 10 (P10), P21, and 4-month-old (4M) *Nox3-Cre*^-/-^;tdTomato^+/+^ (WT) and *Nox3-Cre*^+/+^;tdTomato^+/+^ (*Nox3*-KO) retinae were prepared to compare the percentages of Brn3a-positive (A) and PVALB-positive cells (B) in the ganglion cell layer (GCL) and the number of PVALB-positive cells (B) in the inner nuclear layer (INL), across five images per mouse (with DAPI counterstaining), and graphed in C and D. B, Upper and lower panels in WT and *Nox3*-KO at each time point show images obtained from the same cryostat sections (indicated by magenta rectangles), but acquired under different image acquisition conditions to clearly visualize PVALB-positive cells in the GCL (arrowheads) and INL (yellow arrows), respectively. Percentages of PVALB-positive cells in the GCL increased from P10–P21, and the number of PVALB-positive cells in the INL increased with age (C and D). However, no significant differences were observed between WT and *Nox3*-KO mice using a Student’s t-test (n = 4). ns, not significant. Scale bars: 20 μm. (PNG 2.12 MB)

High Resolution Image (TIF 8.63 MB)

Supplementary figure 7 Thickness of retinae in WT and *Nox3*-KO mice. Cryostat sections of postnatal day 10 (P10), P21, and 2-month-old (2M) *Nox3-Cre*^-/-^;tdTomato^+/+^ (WT) and *Nox3-Cre*^+/+^;tdTomato^+/+^ (*Nox3*-KO) retinae were stained with DAPI for measurement of thickness, shown in A (1 = NFL + GCL + IPL + INL and 2 = NFL+ GCL + IPL + INL + OPL + ONL), and graphed in B. The dots and rhombi in the graphs indicate the number of samples analyzed. No significant differences were observed in either Thickness 1 or Thickness 2 by Student’s t-test. NFL, nerve fiber layer; GCL, ganglion cell layer; IPL, inner plexiform layer; INL, inner nuclear layer; OPL, outer plexiform layer; ONL, outer nuclear layer. (PNG 392 KB)

High Resolution Image (TIF 1.51 MB)

Supplementary figure 8 Apoptosis in the GCL and INL of WT and *Nox3*-KO mice. Whole-mount retinae from postnatal day 21 *Nox3-Cre*^-/-^;tdTomato^+/+^ (WT) and *Nox3-Cre*^+/+^;tdTomato^+/+^ (*Nox3*-KO) mice were prepared for TUNEL assay with DAPI counterstaining. 3D-reconstructed lateral projection images were obtained to identify retinal layers (A). The number of TUNEL-positive cells (yellow arrows) in the ganglion cell layer (GCL) and inner nuclear layer (INL), across five images per mouse (B), showed no significant difference between WT and *Nox3*-KO mice by Student’s t-test (n = 4). ONL, outer nuclear layer. Scale bars: 10 μm. (PNG 305 KB)

High Resolution Image (TIF 1.50 MB)

## Data Availability

This study includes no data deposited in external repositories.
