# Supplementary figures and images for: Nox3 expression and function in retinal ganglion cells and Amacrine cells
Source: Cell Mol Life Sci. 2025 Dec 3;83(1):28. doi: 10.1007/s00018-025-05876-6 (PMC12775227; doi:10.1007/s00018-025-05876-6)

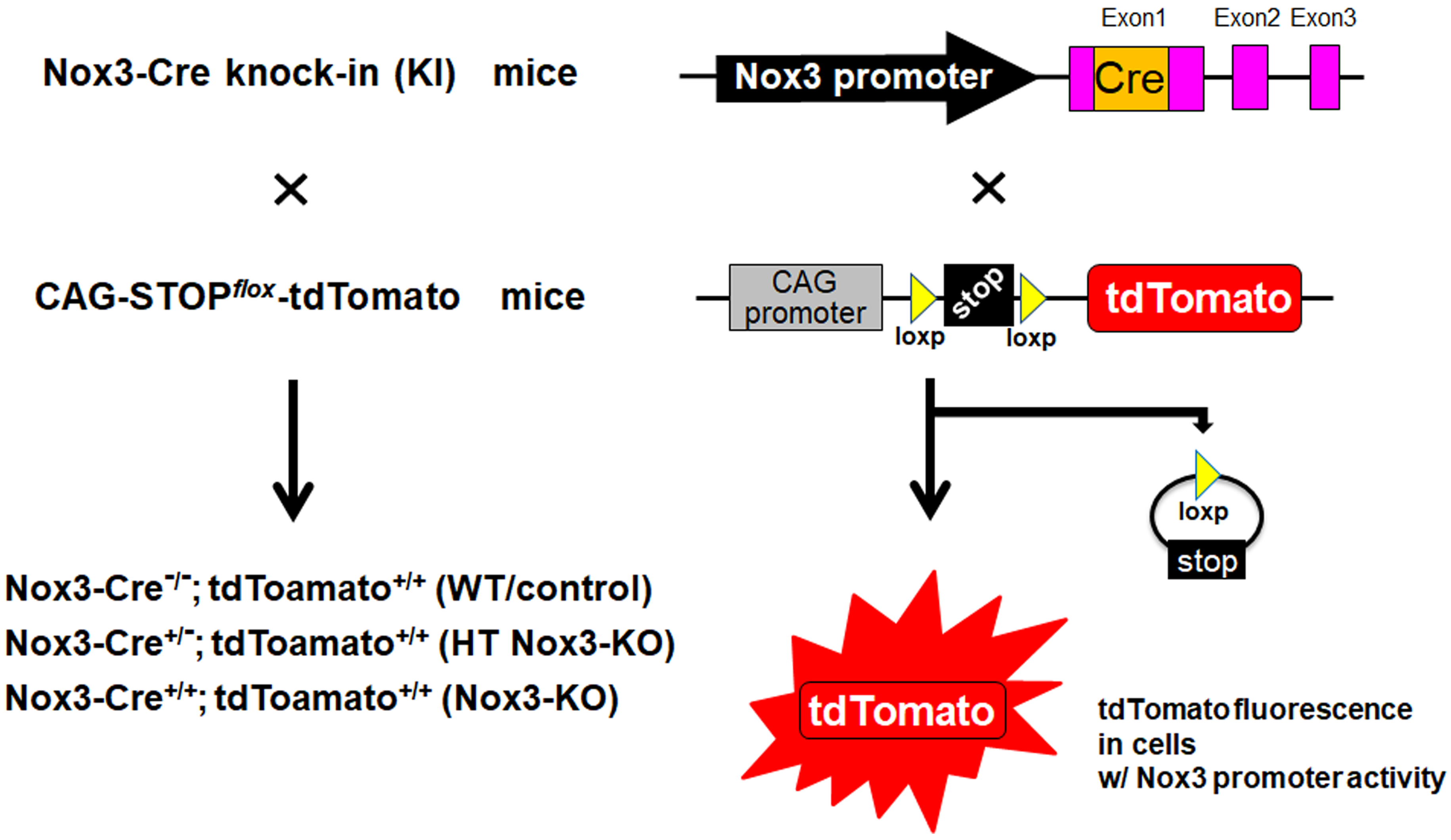

Supplement: Supplementary file 1 — Nox3-Cre;tdTomato mice for detection of Nox3 expression. Illustration depicting the genetic construction of Nox3 mutant mice (Nox3-Cre knock-in [KI]), in which Cre recombinase with a poly(A) sequence was inserted into the ATG site of exon 1 of Nox3. Nox3-Cre KI mice were crossed with CAG-stopflox-tdTomato mice to obtain Nox3-Cre-/-;tdTomato+/+ (WT/control), Nox3-Cre+/-;tdTomato+/+ (heterozygous [HT] Nox3 knockout [KO]), and Nox3-Cre+/+;tdTomato+/+ (Nox3-KO) lines. (PNG 979 KB) [file 18_2025_5876_Fig6_ESM.png]

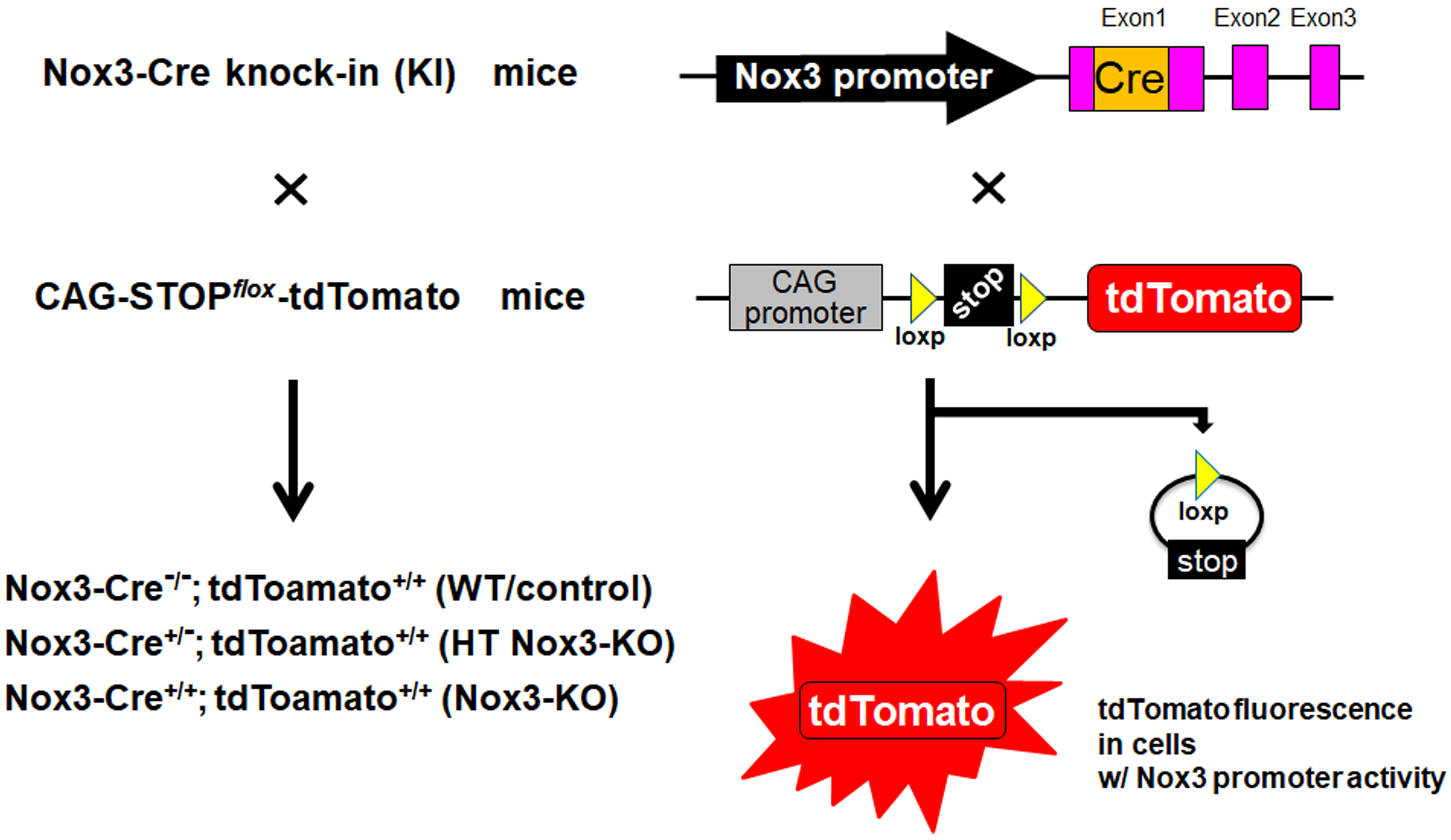

Supplement: Supplementary file 2 — High Resolution Image (TIF 1.66 MB) [file 18_2025_5876_MOESM1_ESM.tif]

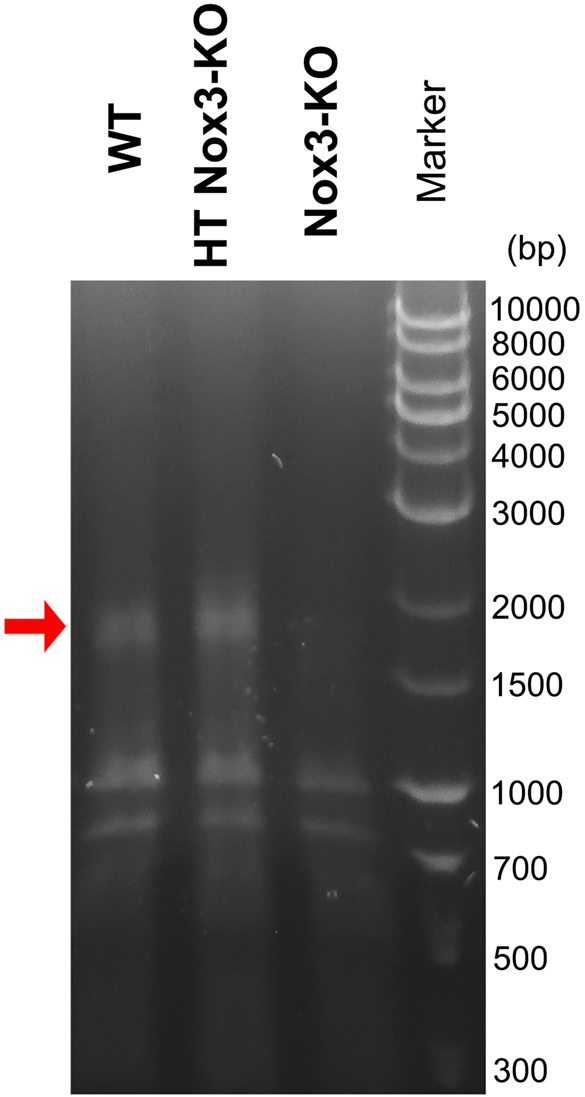

Supplement: Supplementary file 3 — Detection of Nox3 mRNA in WT and HT Nox3-KO, but not in Nox3-KO retinae. Reverse transcription was performed using 2 mg of total RNA from 2-month-old Nox3-Cre-/-;tdTomato+/+ (WT), Nox3-Cre+/-;tdTomato+/+ (HT Nox3-KO), and Nox3-Cre+/+;tdTomato+/+ (Nox3-KO) retinae. PCR using the Nox3-specific primer pair yields 1710 bp bands (including both start and stop codons, indicated by an arrowhead) in WT and HT Nox3-KO, but not in Nox3-KO retinae. (PNG 270 KB) [file 18_2025_5876_Fig7_ESM.png]

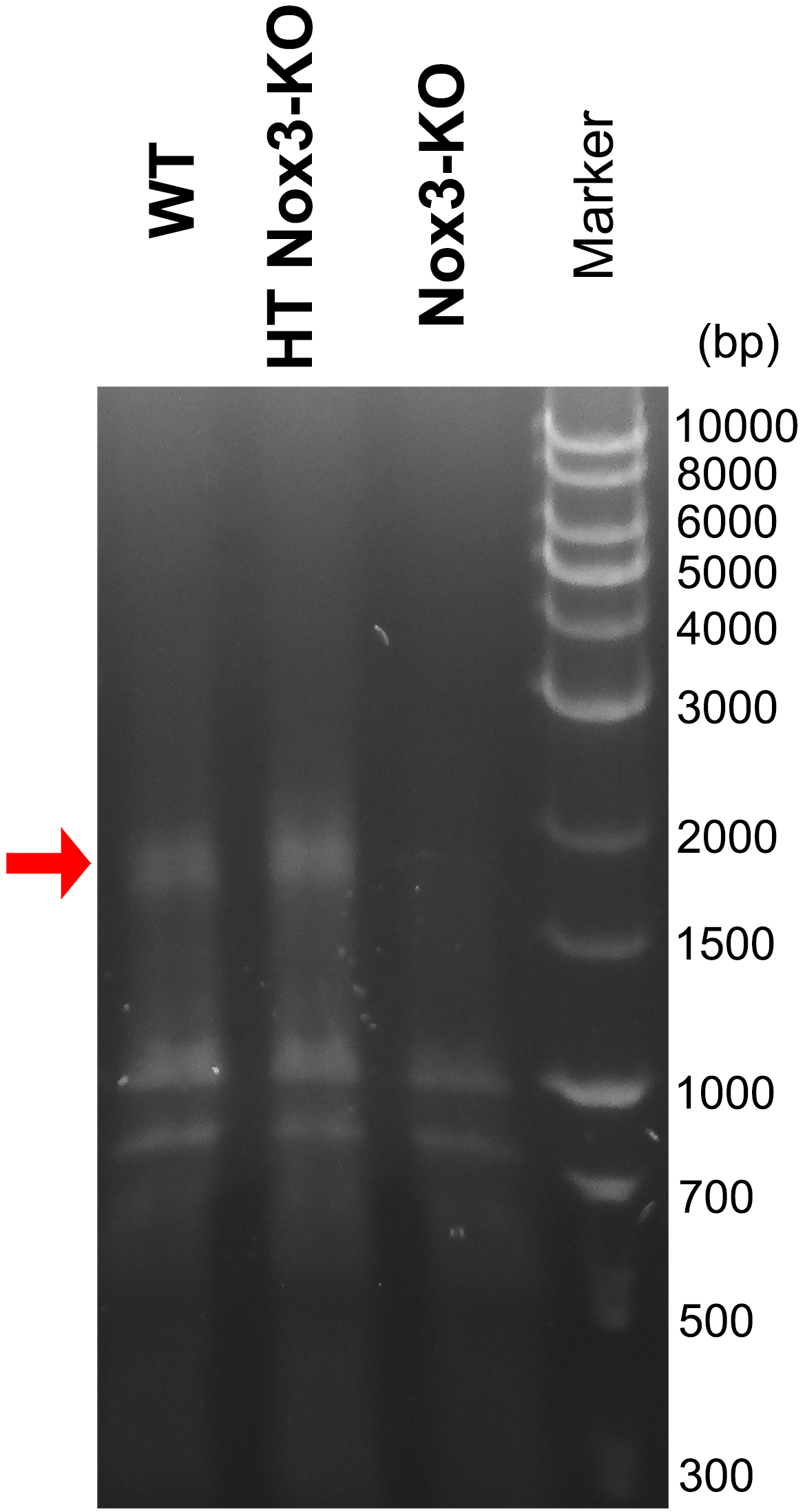

Supplement: Supplementary file 4 — High Resolution Image (TIF 4.55 MB) [file 18_2025_5876_MOESM2_ESM.tif]

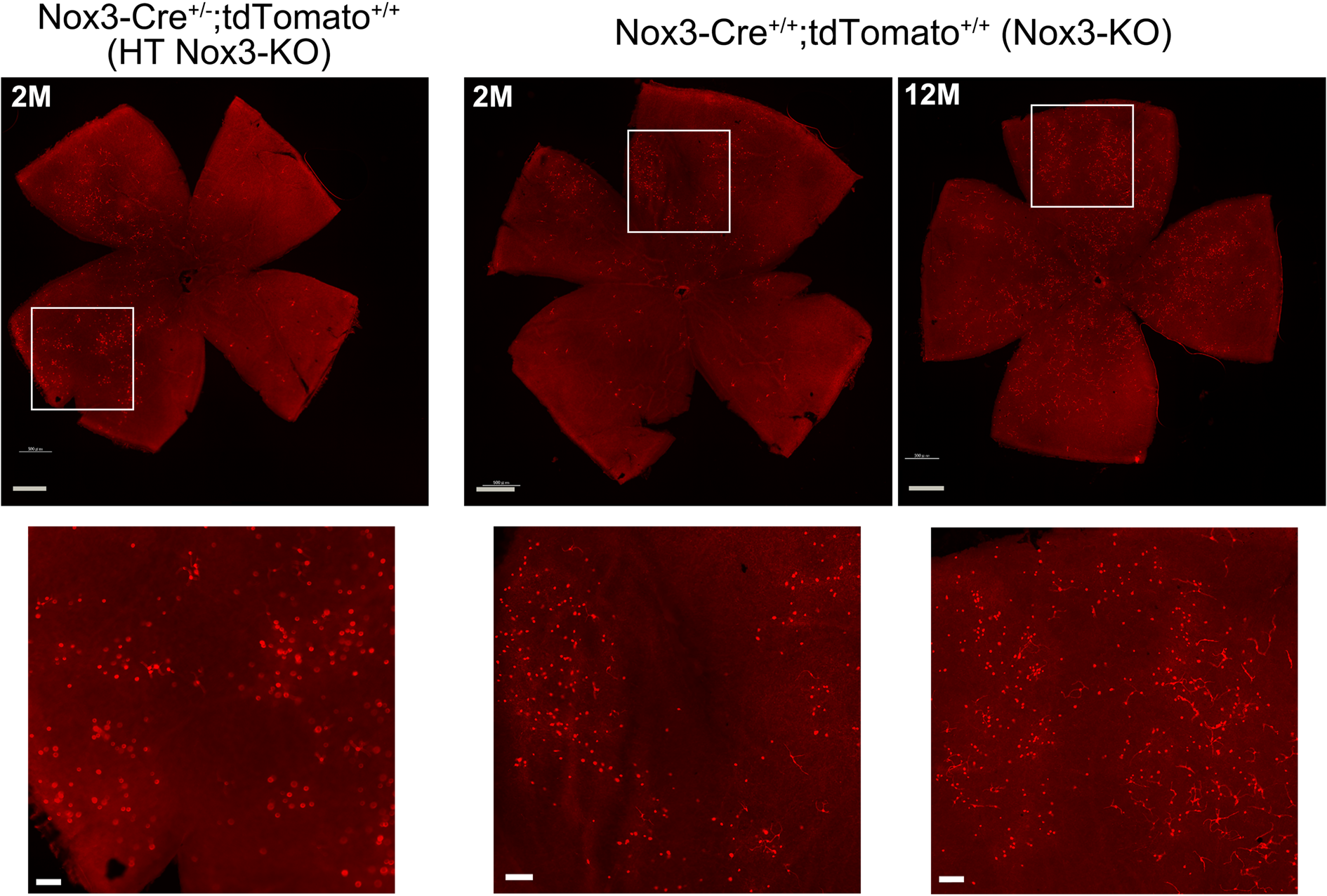

Supplement: Supplementary file 5 — Punctate accumulations of tdTomato-positive cells in the retina.Whole-mount retinae (scale bars: 500 μm) were prepared from 2-month-old (2M) Nox3-Cre+/-;tdTomato+/+(HT Nox3-KO) and 2M and 12M Nox3-Cre+/+;tdTomato+/+ (Nox3-KO) mice. Magnified images (scale bars: 100 μm) indicated by squares are shown. tdTomato-positive cells are not evenly distributed but are clustered in regions in the retina. (PNG 1.29 MB) [file 18_2025_5876_Fig8_ESM.png]

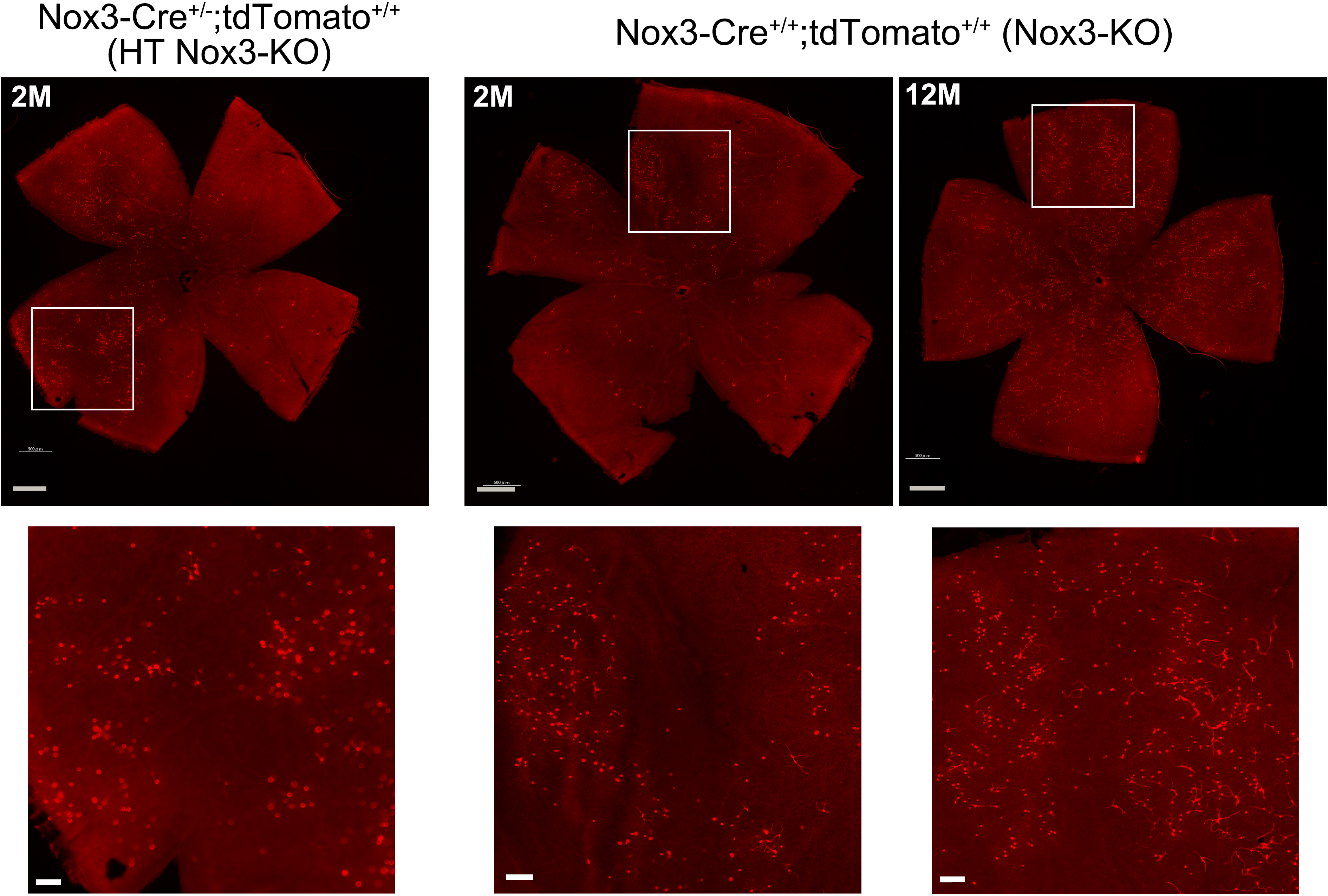

Supplement: Supplementary file 6 — High Resolution Image (TIF 4.78 MB) [file 18_2025_5876_MOESM3_ESM.tif]

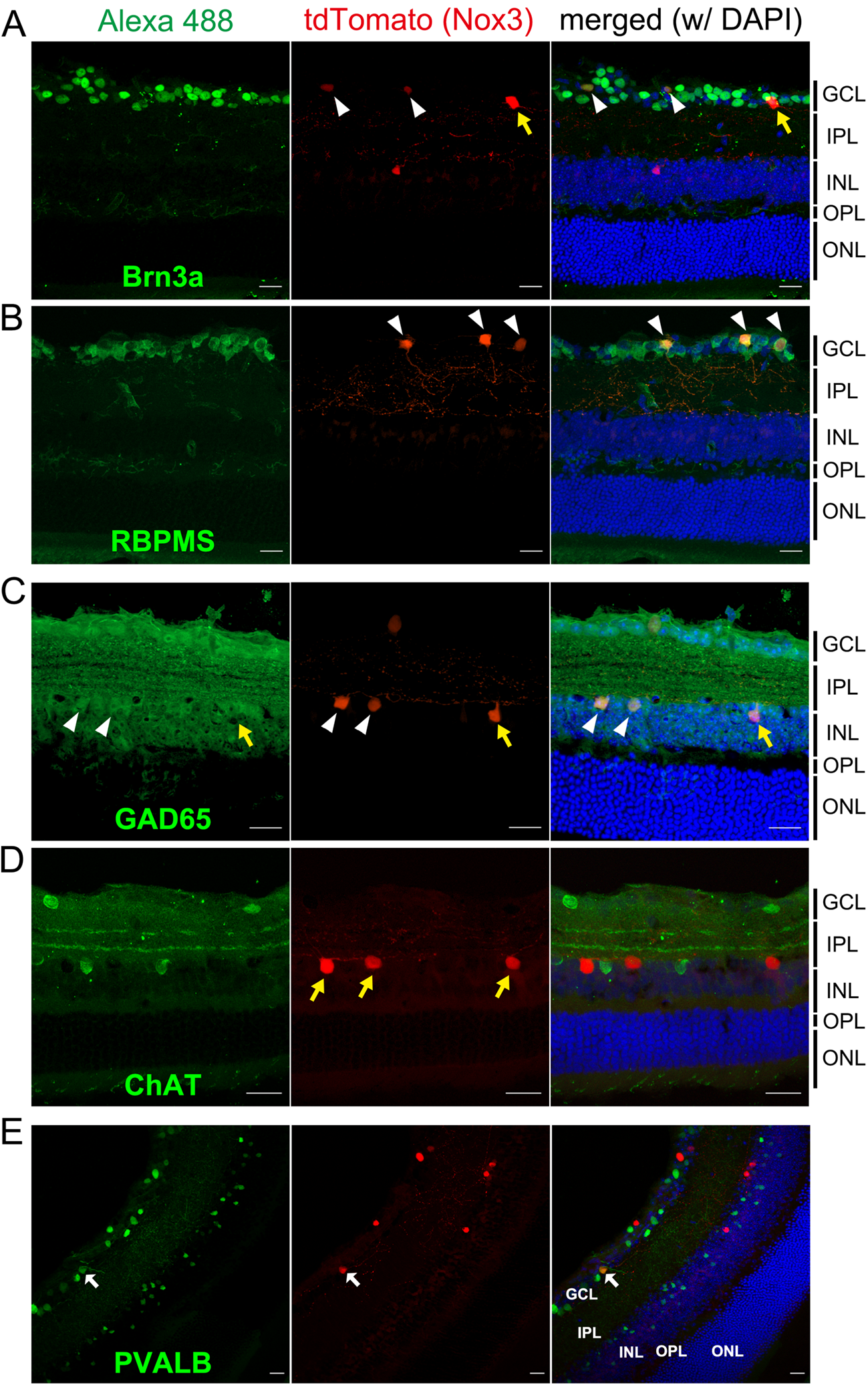

Supplement: Supplementary file 7 — Immuno histological identification of tdTomato-positive cells in the GCL and INL. Cryostat sections of retinae were prepared from Nox3-Cre+/-;tdTomato+/+ (HT Nox3-KO) mice for immunostaining with DAPI counterstaining. tdTomato-positive cells in the ganglion cell layer (GCL) are positive for Brn3a (A, arrowheads) and RBPMS (B, arrowheads), indicating that they are retinal ganglion cells (RGCs). tdTomato-positive cells that are negative for Brn3a (A, yellow arrows) may be displaced amacrine cells (ACs). tdTomato-positive cells in the inner nuclear layer (INL) were positive for GAD65 (C, arrowheads), but not for ChAT (D, yellow arrows), indicating that they are GABAergic ACs. A tdTomato-positive cell that is negative for GAD65 (C, yellow arrows) is detected. tdTomato-positive ACs are primarily negative for PVALB (E), but some cells in the GCL that are positive for both tdTomato and PVALB (E, arrows) were detected. Scale bars: 20 μm. (PNG 2.07 MB) [file 18_2025_5876_Fig9_ESM.png]

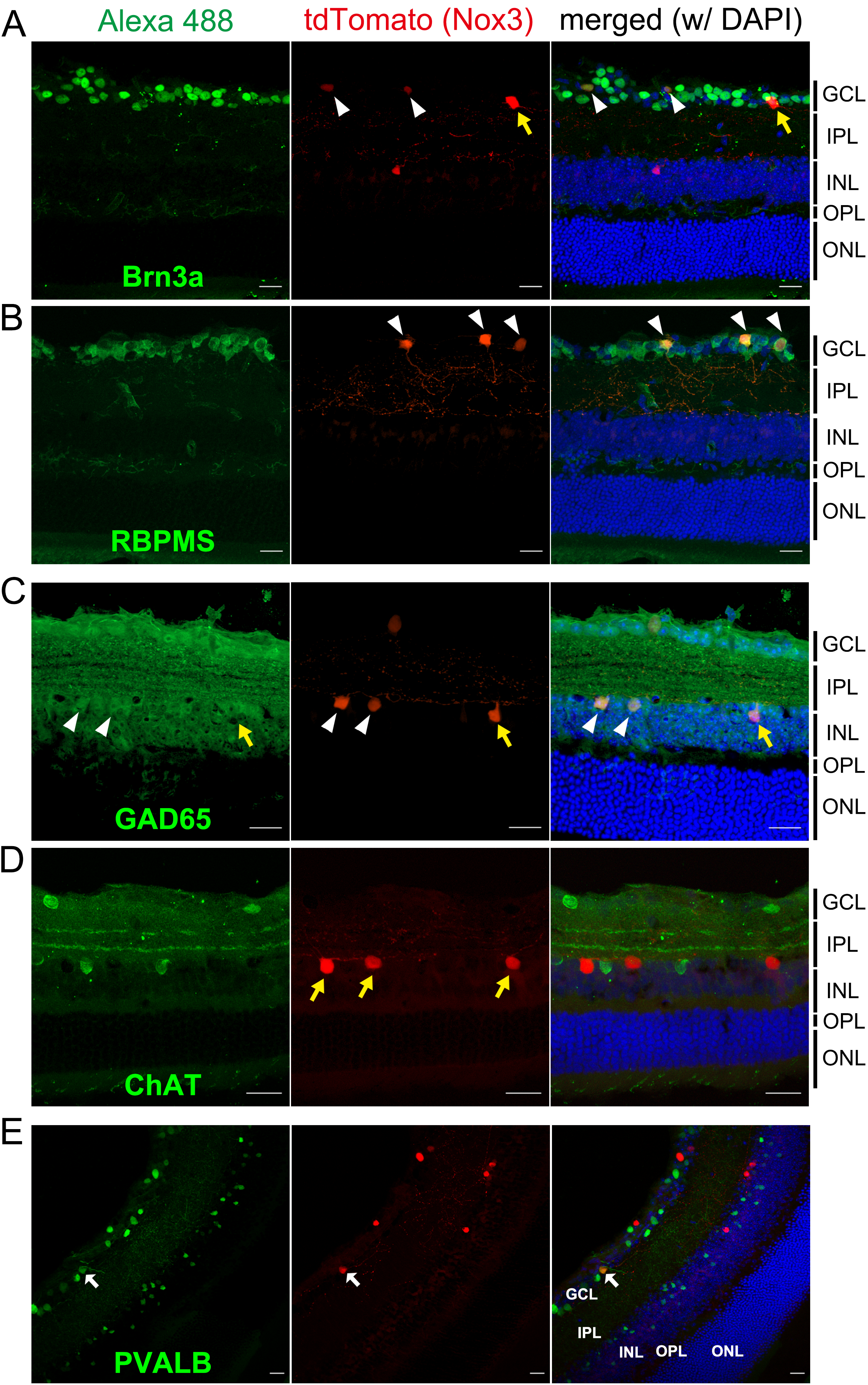

Supplement: Supplementary file 8 — High Resolution Image (TIF 8.53 MB) [file 18_2025_5876_MOESM4_ESM.tif]

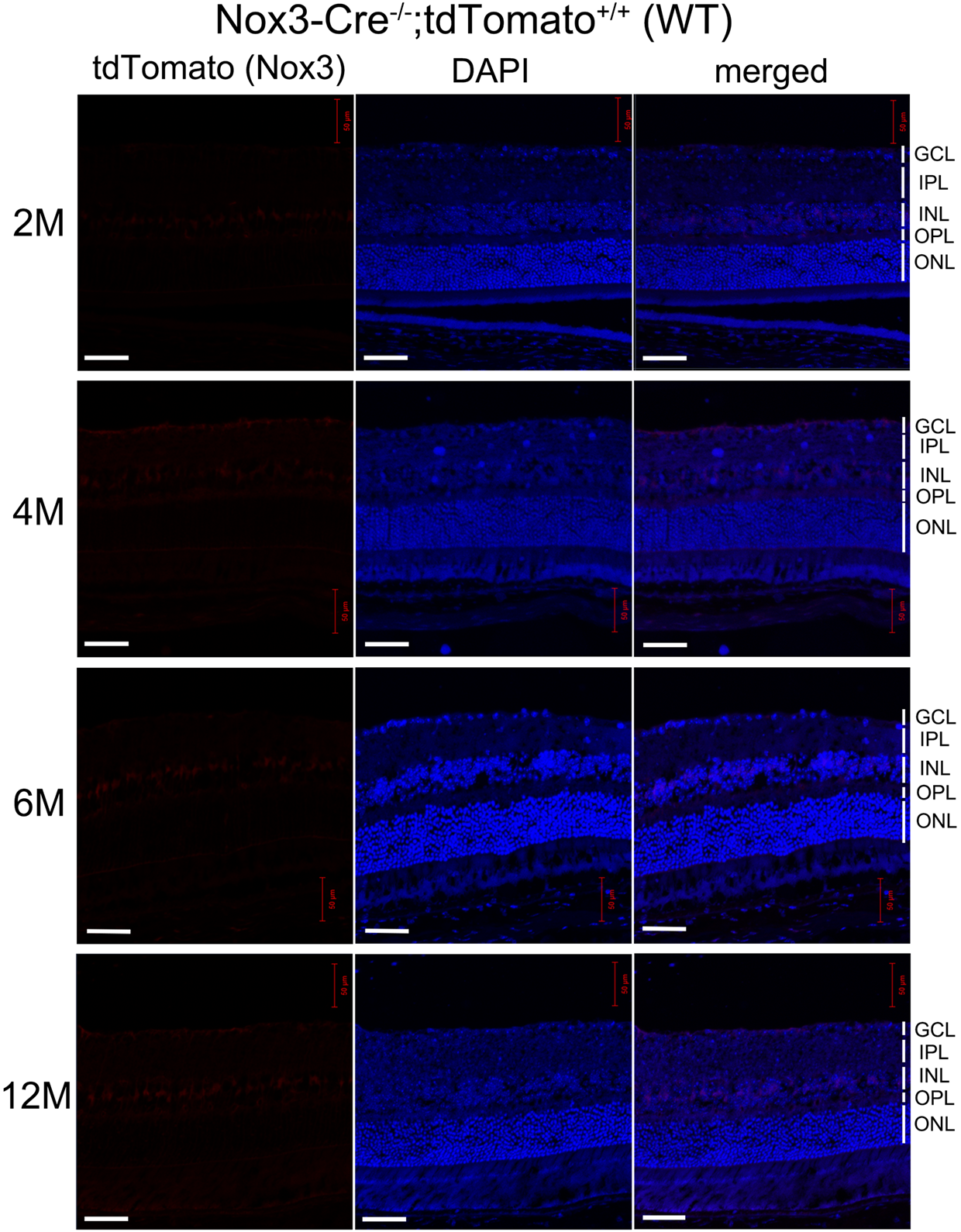

Supplement: Supplementary file 9 — No tdTomato-positive retinal ganglion cells (RGCs) or amacrine cells (ACs) in WT mice.Cryostat sections of 2-month-old (2M), 4M, 6M, and 12M Nox3-Cre-/-;tdTomato+/+ (WT) retinae were stained with DAPI. No tdTomato-positive cells exist in the ganglion cell layer (GCL) or inner nuclear layer (INL) at all ages. IPL, inner plexiform layer; OPL, outer plexiform layer; ONL, outer nuclear layer. Scale bars: 50 μm. (PNG 1.41 MB) [file 18_2025_5876_Fig10_ESM.png]

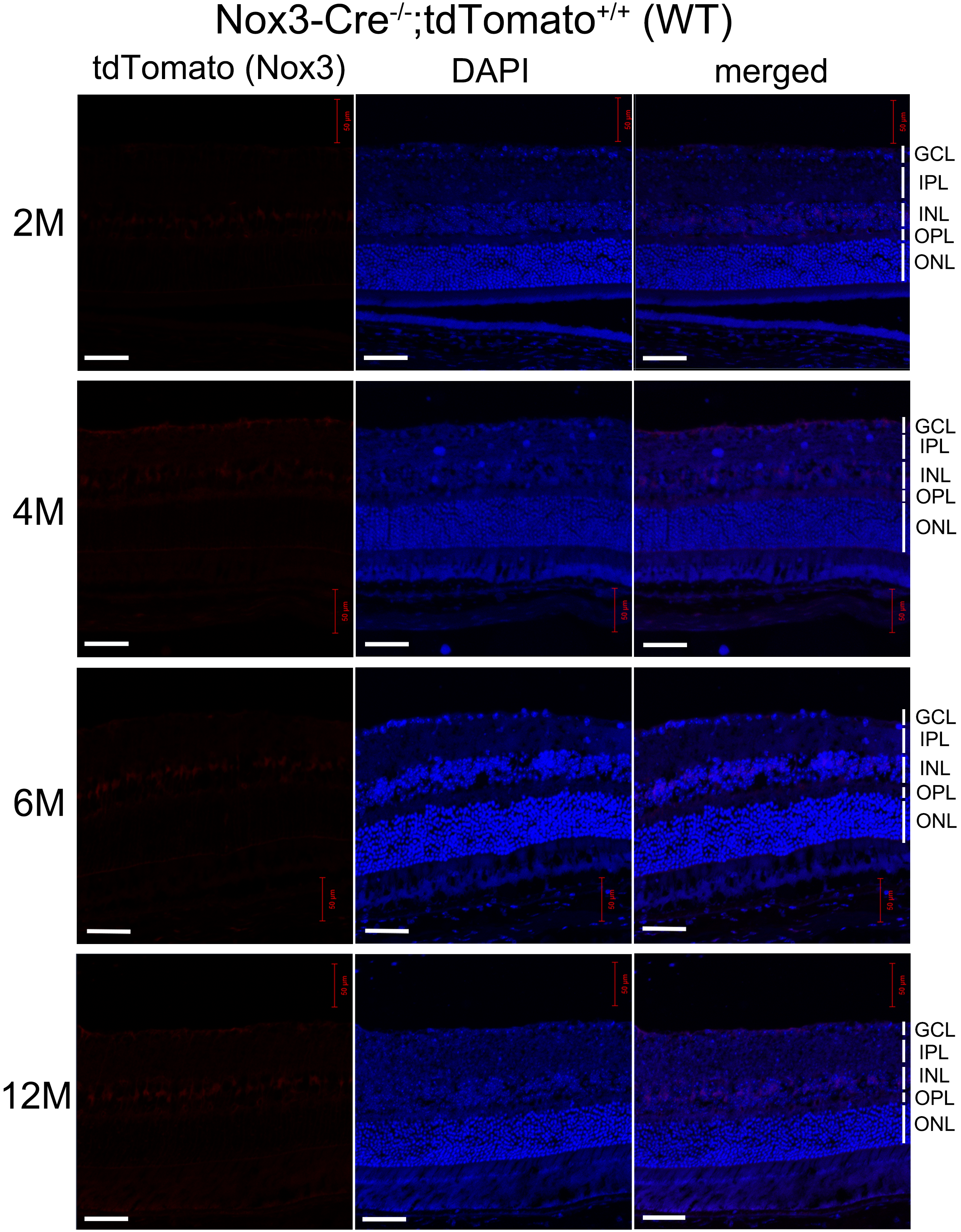

Supplement: Supplementary file 10 — High Resolution Image (TIF 5.64 MB) [file 18_2025_5876_MOESM5_ESM.tif]

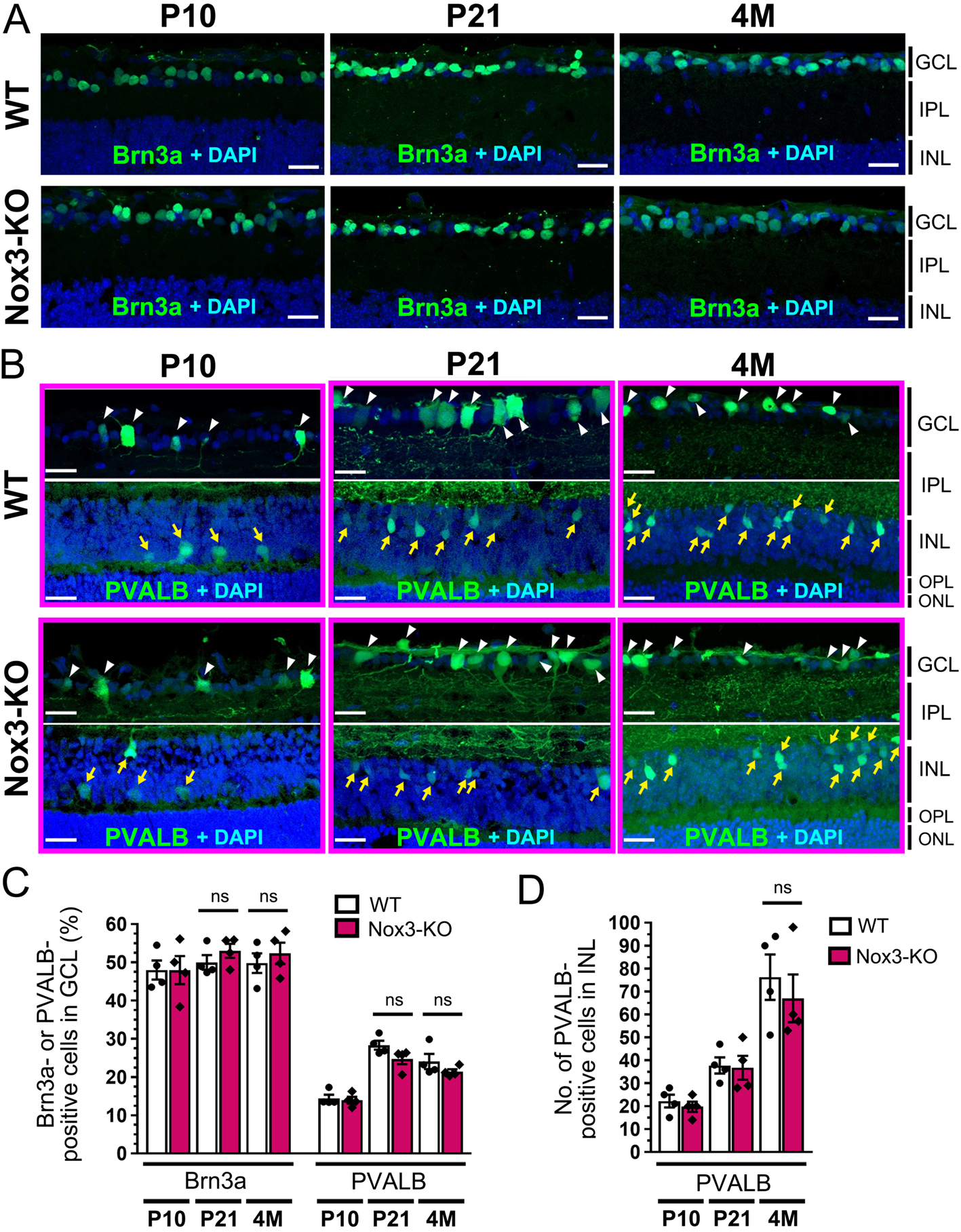

Supplement: Supplementary file 11 — Developmental changes in Brn3a-positive and PVALB-positive cells in WT and Nox3-KO mice. Cryostat sections of postnatal day 10 (P10), P21, and 4-month-old (4M) Nox3-Cre-/-;tdTomato+/+ (WT) and Nox3-Cre+/+;tdTomato+/+ (Nox3-KO) retinae were prepared to compare the percentages of Brn3a-positive (A) and PVALB-positive cells (B) in the ganglion cell layer (GCL) and the number of PVALB-positive cells (B) in the inner nuclear layer (INL), across five images per mouse (with DAPI counterstaining), and graphed in C and D. B, Upper and lower panels in WT and Nox3-KO at each time point show images obtained from the same cryostat sections (indicated by magenta rectangles), but acquired under different image acquisition conditions to clearly visualize PVALB-positive cells in the GCL (arrowheads) and INL (yellow arrows), respectively. Percentages of PVALB-positive cells in the GCL increased from P10–P21, and the number of PVALB-positive cells in the INL increased with age (C and D). However, no significant differences were observed between WT and Nox3-KO mice using a Student’s t-test (n = 4). ns, not significant. Scale bars: 20 μm. (PNG 2.12 MB) [file 18_2025_5876_Fig11_ESM.png]

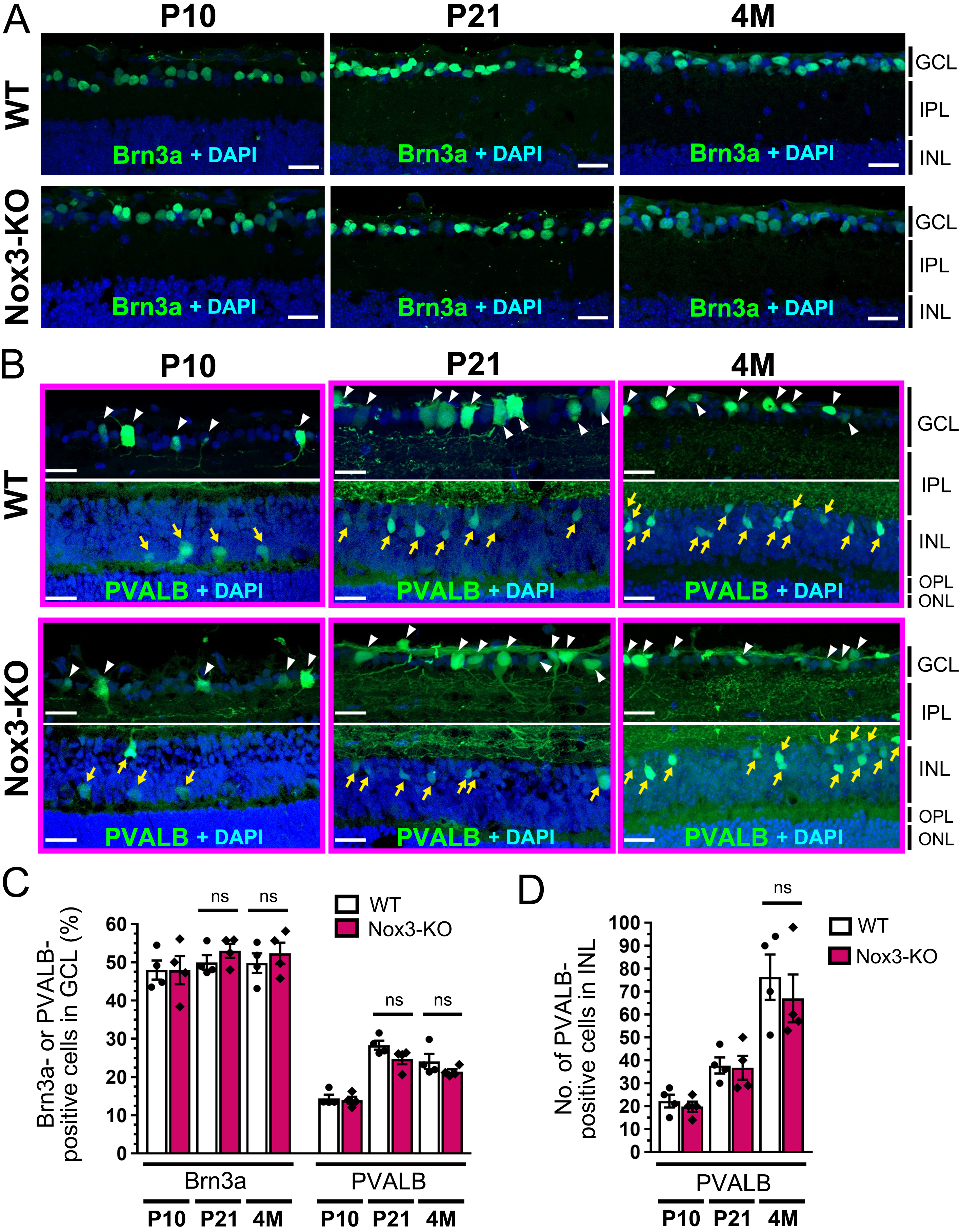

Supplement: Supplementary file 12 — High Resolution Image (TIF 8.63 MB) [file 18_2025_5876_MOESM6_ESM.tif]

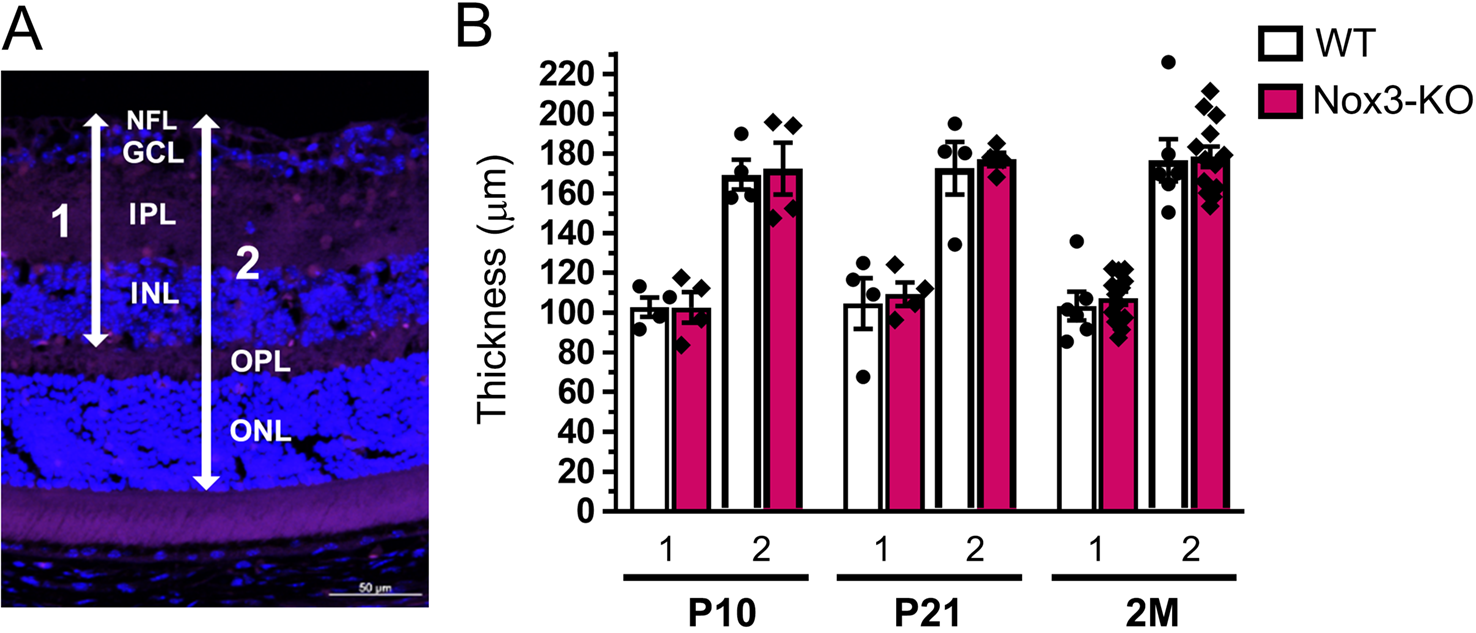

Supplement: Supplementary file 13 — Thickness of retinae in WT and Nox3-KO mice. Cryostat sections of postnatal day 10 (P10), P21, and 2-month-old (2M) Nox3-Cre-/-;tdTomato+/+ (WT) and Nox3-Cre+/+;tdTomato+/+ (Nox3-KO) retinae were stained with DAPI for measurement of thickness, shown in A (1 = NFL + GCL + IPL + INL and 2 = NFL+ GCL + IPL + INL + OPL + ONL), and graphed in B. The dots and rhombi in the graphs indicate the number of samples analyzed. No significant differences were observed in either Thickness 1 or Thickness 2 by Student’s t-test. NFL, nerve fiber layer; GCL, ganglion cell layer; IPL, inner plexiform layer; INL, inner nuclear layer; OPL, outer plexiform layer; ONL, outer nuclear layer. (PNG 392 KB) [file 18_2025_5876_Fig12_ESM.png]

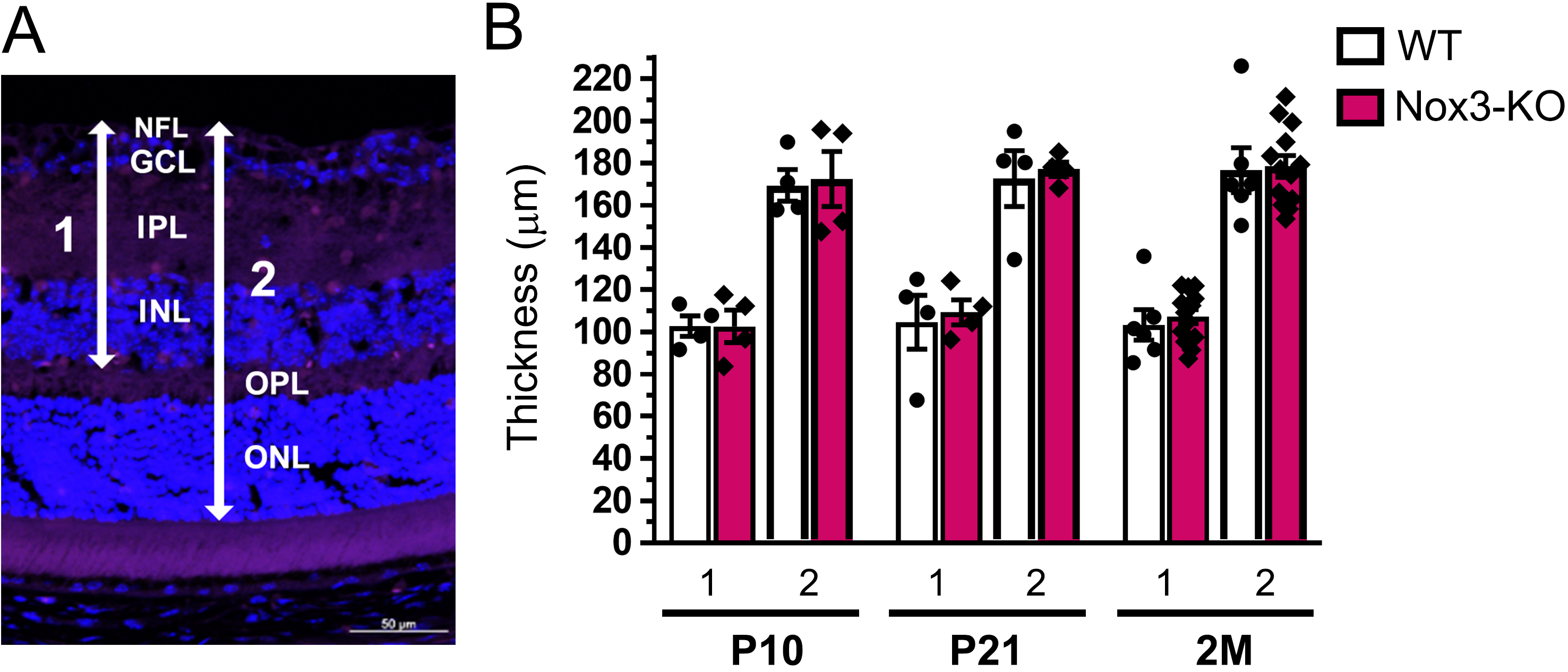

Supplement: Supplementary file 14 — High Resolution Image (TIF 1.51 MB) [file 18_2025_5876_MOESM7_ESM.tif]

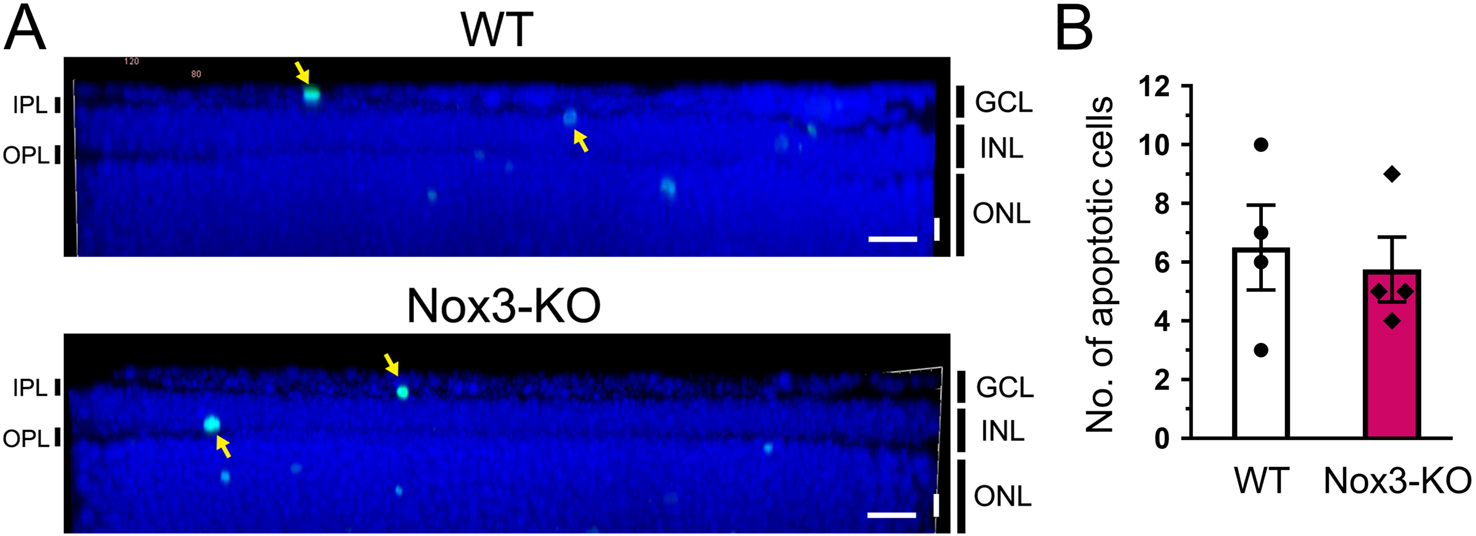

Supplement: Supplementary file 15 — Apoptosis in the GCL and INL of WT and Nox3-KO mice. Whole-mount retinae from postnatal day 21 Nox3-Cre-/-;tdTomato+/+ (WT) and Nox3-Cre+/+;tdTomato+/+ (Nox3-KO) mice were prepared for TUNEL assay with DAPI counterstaining. 3D-reconstructed lateral projection images were obtained to identify retinal layers (A). The number of TUNEL-positive cells (yellow arrows) in the ganglion cell layer (GCL) and inner nuclear layer (INL), across five images per mouse (B), showed no significant difference between WT and Nox3-KO mice by Student’s t-test (n = 4). ONL, outer nuclear layer. Scale bars: 10 μm. (PNG 305 KB) [file 18_2025_5876_Fig13_ESM.png]

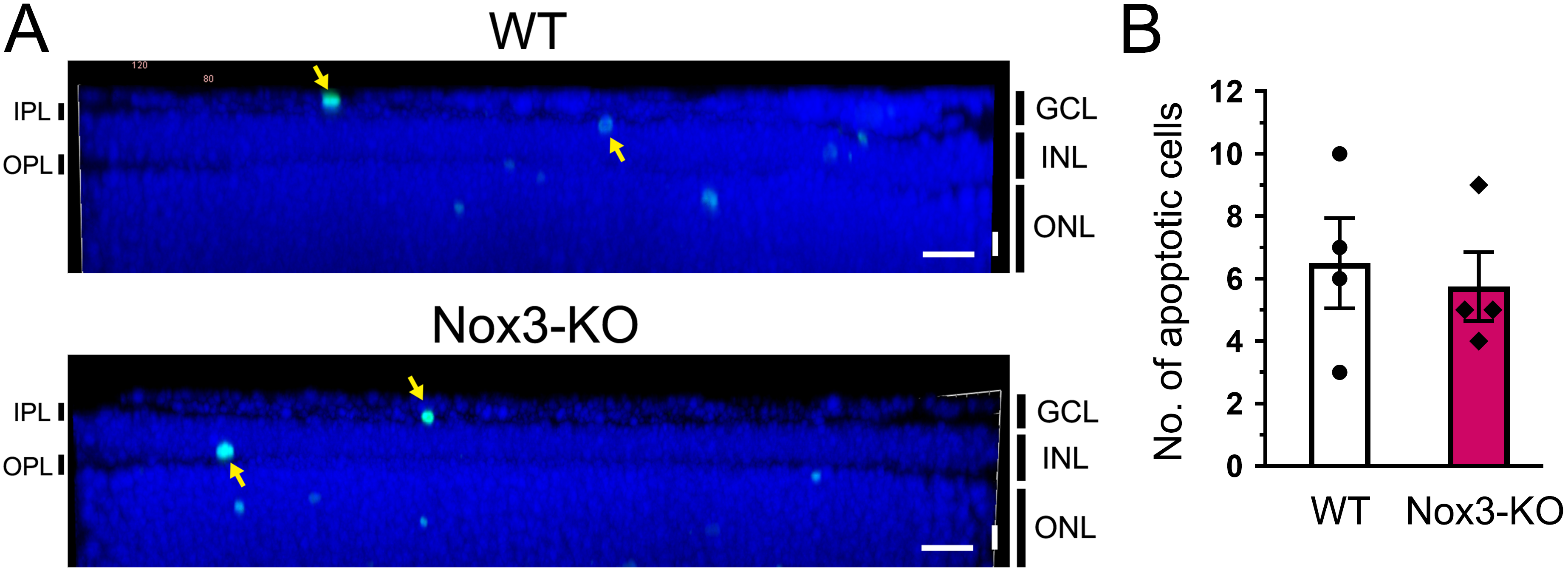

Supplement: Supplementary file 16 — High Resolution Image (TIF 1.50 MB) [file 18_2025_5876_MOESM8_ESM.tif]
